# Manganese-oxidizing *Exiguobacterium acetylicum* 4-3-1 reduces cadmium accumulation in spinach

**DOI:** 10.3389/fmicb.2025.1734825

**Published:** 2026-01-13

**Authors:** Yujia Sun, Mengyao Ding, Wenjuan Zheng, Haoran Zhang, Zhenkun Lu, Jian Zhang, Guoyan Zhao

**Affiliations:** 1 College of Life Science, Shandong Normal University, Jinan, China; 2 College of Resources and Environmental Sciences, China Agricultural University, Beijing, China; 3 College of Geography and Environment, Shandong Normal University, Jinan, China; 4 State Key Laboratory of Biobased Material and Green Papermaking, Qilu University of Technology, Shandong Academy of Sciences, Jinan, China

**Keywords:** cadmium accumulation, manganese-oxidizing bacteria, plant growth-promoting rhizobacteria, sustainable agriculture, synthetic community

## Abstract

Cadmium (Cd) accumulation in edible plants is a significant global concern. This research explores the potential of a manganese-oxidizing rhizobacterium, *Exiguobacterium acetylicum* 4-3-1, to promote spinach growth while reducing Cd uptake. The bacterium produces indole-3-acetic acid and siderophores and effectively removed 73.74% of free CdCl_2_. Under Cd stress (10.5 mg/kg), *E. acetylicum* 4-3-1 significantly increased spinach biomass by 184.3% (dry weight) and chlorophyll content by 33.99%, while decreasing the Cd concentration in spinach leaves by 53.07% through both intrinsic and extrinsic mechanisms. Intrinsically, *E. acetylicum* 4-3-1 inoculation up-regulated pathways related to photosynthesis and energy metabolism in spinach, while down-regulating genes linked to heavy metal transport. Extrinsically, it oxidizes Mn(II) to form manganese oxides that may immobilize Cd. Moreover, inoculation with strain 4-3-1 altered the rhizosphere microbiome of spinach, increasing the presence of beneficial bacteria like Bacillales. A synthetic community (SynCom) composed of *Bacillus subtilis* and *E. acetylicum* 4-3-1 demonstrated synergistic effects on spinach growth under Cd stress. Thus, *E. acetylicum* 4-3-1 has the potential for Cd bioremediation in crops and promotes sustainable agriculture.

## 1 Introduction

Cadmium (Cd) is considered one of the most hazardous heavy metals due to its high mobility within soil–plant systems and its severe toxicity to humans, even at low concentrations, coupled with an exceptionally long biological half-life of 10 to 30 years (Chen et al., 2019). As a non-essential trace element for both plants and animals, Cd is ubiquitous in the environment, with contamination levels reaching 0.3 mg/kg in soils with a pH less than 7.5 and 0.6 mg/kg in soils with a pH greater than 7.5 (Shi-Bao et al., 2018). In China, 19.4% of agricultural soils exceed these Cd contamination levels (Mu et al., 2023), whereas in the European Union, 5.5% of agricultural areas exceed these safety thresholds (Tóth et al., 2016). Japan has reported over 6,000 hectares of land contaminated with Cd (Arao et al., 2010). In the Northern Plains of the United States, Cd concentrations can reach up to 0.4 mg/kg (Jacob et al., 2013), while in the Canadian Great Plains, the median concentration is 0.3 mg/kg (Garrett, 1994). The transfer of heavy metals from soil to plants constitutes a primary pathway for human exposure to soil contamination. Of particular concern is the accumulation of Cd in leafy vegetables cultivated in contaminated soils. A study investigating the differences in Cd accumulation among 32 vegetable varieties found that the Cd content ranged from 0.01 to 0.24 mg·kg^−1^ (Mu et al., 2023). Among the vegetables, spinach (*Spinacia oleracea*) is recognized for its ability to accumulate high levels of Cd (367.7 mg·kg^−1^) in its edible leaves (Filipa et al., 2020). It is regarded as a Cd-tolerant crop and is utilized for phytoremediation purposes (Salaskar et al., 2011); however, it is also one of the most widely consumed vegetables globally (Zhao et al., 2025), raising significant concerns regarding global food safety.

The accumulation of Cd in plants is regulated by both internal genetic factors and extrinsic microbial interactions. Internally, no specific transporter for Cd has been identified in plants. The uptake of Cd appears to be linked to the transport pathways of other elements, including zinc and manganese (Mn) (Luo et al., 2018; Yan et al., 2019). For instance, in rice, the OsNRAMP5 protein acts as a Mn/Cd co-transporter, moving Cd from the rhizosphere into the cytoplasm, with its polar localization on the distal side of the plasma membrane in the root exodermis and endodermis (Peng et al., 2008; Tang et al., 2022). Additionally, the up-regulation of the vacuolar sequestration protein OsHMA3 enhances the compartmentalization of Mn within root cell vacuoles and competitively inhibits the translocation of Cd to the aerial parts of the plant (Chen et al., 2023; Zhang et al., 2023).

To tackle soil cadmium pollution, various remediation strategies have been developed, yet each has its limitations. Traditional physicochemical methods have disadvantages such as extensive construction requirements, high expenses, potential secondary pollution risks, and considerable alteration of soil’s physical and chemical properties (Deng et al., 2024). Phytoremediation, using hyperaccumulator plants, is more eco-friendly but has long remediation cycles, limited biomass, and is not compatible with conventional agriculture. Recently, plant-microbe combined remediation has shown promise by introducing exogenous functional microbial agents to enhance the efficiency of hyperaccumulator plants (Chi et al., 2025), or through passivation using abiotic materials such as carbon nanotubes (Chen et al., 2023). However, these methods still commonly face challenges such as reliance on specific plant species, have complex environmental interactions, and offer limited protection to staple crops. Therefore, it is of urgent significance to develop a novel microbial remediation strategy that can be directly applied to crops, operates independently of hyperaccumulator plants, and has clearly defined mechanisms, in order to ensure agricultural product safety.

In this context, utilizing beneficial rhizosphere microorganisms as independent “bio-inoculants” to directly enhance crop stress resistance and reduce heavy metal uptake is an appealing alternative approach. These microorganisms can interact with Cd through various mechanisms, including biosorption, bioprecipitation, and bioaccumulation (Li et al., 2019). They sequester Cd ions on their cell surfaces or within extracellular polymeric substances, effectively immobilizing the metal and reducing its uptake by plants (Deo et al., 2024; Hassan et al., 2013). Some microbes can alter the chemical speciation of Cd through redox transformations or precipitation, converting it into less soluble and less toxic forms (Li et al., 2024; Hu et al., 2011). Furthermore, their metabolic activities can modify soil properties, such as pH and redox potential, which in turn govern the solubility and bioavailability of Cd (Sardans et al., 2023); Beyond direct interactions with cadmium (Cd), Plant Growth-Promoting Rhizobacteria (PGPR) can indirectly facilitate remediation by enhancing plant growth and tolerance to metal stress. By producing phytohormones such as indole-3-acetic acid (IAA), siderophores, and other beneficial compounds, PGPR promote root and shoot development, thereby enabling plants to more effectively endure the toxic effects of Cd (Pramanik et al., 2018; Tak et al., 2013).

Manganese-oxidizing bacteria, which are widely distributed in natural environments, have emerged as a novel functional group for regulating the bioavailability of heavy metals due to their unique biomineralization capabilities (Madison et al., 2013; Daye et al., 2019). The interaction mechanisms between manganese-oxidizing bacteria and plants remain unclear; however, studies suggest that these bacteria may establish mutualistic relationships with plants. For instance, epiphytic and endophytic bacteria exhibiting manganese-oxidizing activity have been identified on plant root surfaces and can enhance the oxidation of iron and manganese through biological oxidation reactions, resulting in the formation of iron-manganese plaques on the root surface (Zhao et al., 2019). This process facilitates the adsorption and fixation of heavy metals in the soil, transforming them into a more stable form and reducing their toxicity to plants (Huang et al., 2022; Liang et al., 2022; Wei et al., 2021). Compared to iron-oxidizing bacteria, another microorganism involved in the formation of iron-manganese plaques on plant root surfaces, manganese-oxidizing bacteria are more suitable for application under neutral to weakly alkaline conditions (pH 6–8). The biogenic manganese oxides (BioMnOx) they produce exhibit a higher adsorption capacity for Cd, with a specific surface area reaching 200–300 m^2^/g (Liu et al., 2024). Additionally, they form a stable structure through interlayer embedding, making them suitable for long-term remediation (Liu et al., 2024). However, few studies have investigated the mechanisms by which manganese-oxidizing bacteria influence plant growth and Cd accumulation, and the molecular mechanisms underlying their effects on plants remain unclear.

Here, we discovered a manganese-oxidizing bacterium in the rhizosphere that enhances spinach growth and reduces Cd uptake. This bacterium modifies root microbial communities and influences spinach gene expression under Cd stress, potentially affecting Cd bioavailability and plant translocation. It shows promise for Cd bioremediation and agricultural applications.

## 2 Materials and methods

### 2.1 Isolation and identification of Cd-removing manganese-oxidizing bacteria from rhizosphere soil

Samples of *Suaeda sals*a were collected from an oil and gas site located in Dongying City, Shandong Province, China (coordinates: 117°87′ ~ 119°26′E, 37°70′ ~ 38°05’N). The root soil was prepared by first removing loosely adhering soil through gentle shaking of the root system to eliminate bulk soil. Subsequently, the tightly adhering soil, referred to as rhizospheric soil, was carefully removed by brushing the root system with sterile brushes. This soil was collected on sterile trays and mixed with sterile water to create an inoculum. This inoculum was then added at a 10% concentration to Luria-Bertani (LB) medium supplemented with 5 mM MnCl_2_ and incubated at 30 °C for 72 h. The LB medium was composed of 10 g/L tryptone, 5 g/L yeast extract, and 10 g/L NaCl, with a pH of 7.0. The resulting bacterial suspension was diluted to concentrations ranging from 10^−3^ to 10^−5^, spread onto LB solid medium containing 5 mM MnCl_2_, and incubated at 30 °C. Repeated streaking techniques were employed to isolate and purify bacterial colonies.

To identify manganese-oxidizing bacteria, we employed the Leukoberbelin blue (LBB) method to quantify the manganese oxidation capability of the strains. The formation of high-valent manganese oxides was indicated by a color change, characterized by an absorption peak at 620 nm (Krumbein and Altmann, 1973). Using these methods, we successfully isolated a bacterium designated as 4-3-1, which demonstrated manganese oxidation activity. The 16S ribosomal RNA (16S rRNA) gene was amplified via polymerase chain reaction (PCR) and subsequently sequenced (Twigg et al., 2018). The resulting sequences were analyzed using NCBI BLASTN1^1^ and EZ-Biocloud.^2^ A maximum-likelihood phylogenetic tree was constructed using MEGA 5.0, with bootstrap values derived from 1,000 resampling iterations (Labella et al., 2018).

By analyzing its morphological, physiological, and biochemical traits, this strain was further identified. A scanning electron microscope (SEM, HITACHI SU8100) was used to examine the morphological characteristics of the strain. The optimal pH, temperature, and NaCl content for the growth of strain were established. Different temperatures (15 °C, 20 °C, 25 °C, 30 °C, and 37 °C) were used for the growth experiments. With one pH unit increments, the pH range for growth was examined between 4.0 and 10.0. Furthermore, several concentrations of NaCl (0, 1, 3, 4, 6, 8, 9, 12, 15, and 20%) were used to assess growth in LB medium. The minimum inhibitory concentration (MIC) of the strain 4-3-1 against MnCl_2_ and CdCl_2_ was then ascertained. The strain was cultured in LB liquid medium for 12 h, with MnCl_2_ concentrations ranging from 0 to 30 mM and CdCl_2_ concentrations ranging from 0 to 0.3 mM. If the optical density at 600 nm (OD_600 nm_) is greater than 0.1, it indicates positive growth. Additionally, the cadmium removal capability of strain 4-3-1 was assessed. This strain was cultivated in LB medium supplemented with 0.05 mM CdCl_2_. After 24 h, the supernatant was collected via centrifugation, and the Cd content was quantified using inductively coupled plasma optical emission spectrometry (ICP-OES, PerkinElmer Avio 200).

### 2.2 Plant growth and co-cultivation with the strain 4-3-1

Spinach (*Spinacia oleracea* L.), a commonly cultivated leafy vegetable, exhibits a notable propensity for Cd uptake due to its capacity to absorb and translocate toxins from the soil to its consumable tissues (Zhao et al., 2025). For our study, we selected the Yinong Super Spinach 398 variety due to its characteristics of disease resistance, heat tolerance, rapid growth, and high yield, which contribute to its widespread cultivation across China. The cultivation protocol began with an initial seed treatment, which involved soaking the seeds in a 2% sodium hypochlorite (NaClO) solution for 15 min, followed by rinsing in distilled water for 2 h. Seeds were then placed on moist, sterile filter paper and incubated in darkness at 30 °C until root lengths reached 0.5 cm. Subsequently, seedlings were sown in nutrient-rich soil until reaching a height of 3–4 cm, after which they were transplanted into designated soil conditions. The experimental soils were categorized into normal and Cd-contaminated soils, with the latter containing 10.5 mg/kg of CdCl_2_. All experimental groups were cultivated under controlled conditions of 25 °C, 60% relative humidity, and illumination of 5,000 Lux.

For the co-cultivation of spinach with the strain 4-3-1, a bacterial suspension was prepared by initially culturing 40 mL of the strain 4-3-1 in LB medium until an OD600 value of 1.0 was achieved. Subsequently, this culture was centrifuged and the cells were resuspended in 100 mL of sterilized 50% Hoagland’s plant nutrient solution (as described by Hoagland and Arnon, 1950) for irrigation purposes. The Hoagland solution contains 506 mg KNO_3_, 945 mg Ca(NO_3_)_2_, 80 mg NH_4_NO_3_, 241 mg MgSO_4_, 136 mg KH_2_PO_4_, 36.7 mg FeNaEDTA, 22.3 mg MnSO_4_, 8.6 mg ZnSO_4_, 6.2 mg H_3_BO_3_, 0.83 mg KI, 0.25 mg Na_2_MoO_4_, 0.025 mg CuSO_4_ and 0.025 mg CoCl_2_. Irrigation was conducted at a frequency of once every 2 days. The uninoculated control group was maintained with 100 mL of sterilized 50% Hoagland’s plant nutrient solution devoid of bacterial inoculation.

### 2.3 Construction of synthetic bacterial communities with the strain 4-3-1 and *Bacillus subtilis*

The *Bacillus subtilis* strain (CICC 10155) was cultured at 28 °C in a nutrient broth medium (pH7.0) composed of 5.0 g of peptone, 3.0 g of beef extract powder, 5 mg of MnSO_4_·H_2_O, and 5.0 g of NaCl per liter. In contrast, strain 4-3-1 was grown at 30 °C in LB medium. Both strains were cultured until they reached an OD600 of 1.0. To evaluate the antagonistic interactions between the indicator strains, the plate confrontation method was employed. This involved restreaking and co-culturing both strains on LB agar at 28 °C for 24 h.

To investigate the growth-promoting and detoxification potential of the synthetic community comprising *Bacillus subtilis* CICC 10155 and *Exiguobacterium acetylicum* 4-3-1 on spinach, bacterial suspensions were prepared by mixing equal volumes of each bacterial solution, both having an OD600 of 1.0. The co-inoculation of this synthetic community with spinach was conducted in accordance with the previously described procedures.

### 2.4 Detection of Cd content in spinach leaves

To examine Cd accumulation in spinach leaves, circular samples with a diameter of 1 cm were collected and stained using a 5 μM concentration of Leadmium™ Green AM dye (Invitrogen, Carlsbad, CA, United States). The stained samples were subsequently placed in 15 mL centrifuge tubes and subjected to vacuum treatment three to four times at −25 °C using a vacuum freeze-dryer. Observations were conducted utilizing a microscope imaging system (Lumazone PyLoN1300B, Teledyne Princeton Instruments). For the quantitative analysis, sterile filter paper was employed to absorb surface moisture from spinach leaves exposed to Cd stress. The leaves were subsequently placed in a constant temperature incubator at 37 °C for 24 h for drying. Following drying, the leaves were ground into a powder using a mortar. Each 0.5 g sample was microwave-digested, and its Cd content was measured using ICP-OES (Avio 200, PerkinElmer, US) by the certified Chengdu Shiji Meiyang Technology Co., laboratory.

### 2.5 Scanning electron microscopy energy-dispersive spectroscopy

The morphological characteristics of biogenic manganese oxides (BioMnOx) were investigated utilizing a Thermo Scientific Apreo 2C scanning electron microscope, operated at an accelerating voltage of 15 kV and achieving a resolution of 1.0 nm. Elemental analysis of the manganese oxidation product, specifically targeting manganese (Mn), carbon (C), and oxygen (O), was conducted using an OXFORD ULTIM Max 65 energy dispersive spectrometer.

### 2.6 X-ray diffraction

The dried BioMnOx sample was subjected to analysis utilizing a Rigaku Ultima IV diffractometer equipped with a copper (Cu) target. The operational parameters were set at a voltage of 40 kV and a current of 40 mA. The scanning procedure was executed with a step size of 0.02° over a range from 5° to 90°, facilitating the phase composition analysis of the BioMnOx produced by *E. acetylicum* 4-3-1.

### 2.7 X-ray photoelectron spectroscopy

X-ray photoelectron spectroscopy (XPS) analyses were conducted using a ThermoFisher ESCALAB250Xi spectrometer. The primary experimental parameters included a vacuum chamber pressure of 1 × 10^−10^ Torr, a resolution of 0.4%, an electron gun spot size of 75 nm, a sensitivity of 1 Mcps, an angular resolution ranging from 5° to 90°, an energy resolution of 0.5 eV, and a sensitivity of 255 KCPS. XPS survey spectra (ranging from 1,100 to 0 eV) and high-resolution narrow scans of carbon (C), oxygen (O), and manganese (Mn) were systematically acquired.

### 2.8 Exploring the physiological and biochemical characteristics of spinach

Spinach plants were harvested after 23 to 25 days. Measurements of root length, plant height, fresh weight, and dry weight were taken for various treatments. Plants were carefully removed from the pots, and the roots were gently rinsed with distilled water. The root and stem lengths were measured and the fresh weight was recorded with an electronic balance. The spinach samples were then dried at 60 °C for 48 h before determining the dry weight. The chlorophyll content in spinach leaves was quantified using an extraction method involving an acetone-ethanol mixture, as referenced in Porra et al. (1989). The concentration of soluble proteins was assessed via the Coomassie Brilliant Blue assay (Bradford, 1976). Soluble sugar extraction and quantification were performed using the anthrone-sulfuric acid method (DuBois et al., 1956). The malondialdehyde (MDA) content was measured by the thiobarbituric acid (TBA) method (Heath and Packer, 1968). Betaine content was determined according to the method described in reference (Grieve and Grattan, 1983). Proline content was assessed using the acidic ninhydrin colorimetric assay (Bates et al., 1973). The activities of Superoxide Dismutase (SOD) and Peroxidase (POD) were evaluated using the Superoxide Dismutase Activity Detection Kit (BC0170, Solarbio) and the Peroxidase Activity Detection Kit (BC0090, Solarbio), respectively. Each experimental group was analyzed in triplicate to ensure reliability.

### 2.9 Metagenomic sequencing analysis

A total of 0.2 g of soil material from Cd-stressed spinach was utilized for the extraction of total genomic DNA using the E.Z.N.A.^®^ Soil DNA Kit, following the manufacturer’s protocol. The concentration and purity of the extracted DNA were determined using the SynergyHTX and NanoDrop2000 instruments, respectively. The quality of the DNA was further assessed via electrophoresis on a 1% agarose gel. DNA fragmentation to an average size of approximately 350 base pairs was achieved using the Covaris M220 (Gene Company Limited, China) to facilitate paired-end library construction. The paired-end library was constructed using the NEXTFLEX Rapid DNA-Seq kit (Bioo Scientific, Austin, TX, United States). Sequencing was conducted on the Illumina NovaSeq™ X Plus platform (Illumina Inc., San Diego, CA, United States) in Majorbio Bio-Pharm Technology (Majorbio Bio-Pharm Technology Co., Ltd., Shanghai, China). The sequencing data underwent processes of splitting, quality filtering, and impurity removal. The refined sequence data were subsequently employed for assembly, contig construction, and gene prediction.

Utilizing the LEfSe tool (available at http://galaxy.biobakery.org/), linear discriminant analysis was performed to detect significantly different bacterial species in spinach soil samples under Cd stress. The PPM abundance method was applied with an LDA threshold > 2, adopting a one-against-all comparison strategy. Biomarker species were examined across taxonomic levels ranging from phylum to genus. The amino acid sequences from the non-redundant gene catalog were aligned to the NCBI NR database for taxonomic annotation using Diamond (version 0.8.35) with an e-value threshold of 1e^−5^ (Buchfink et al., 2015; http://www.diamondsearch.org/index.php). Gene function annotation was performed by a BLASTp search (threshold e-value 10^−5^) against the Cluster of Orthologous Genes (COG) database and KEGG database.^3^

### 2.10 Dual transcriptome sequencing (dual RNA-seq) analysis

Spinach leaves co-cultivated with the strain 4-3-1 under cadmium (Cd) stress (10.5 mg/Kg) for 25 days were analyzed via dual RNA sequencing at Majorbio Bio-Pharm Technology Co., Ltd. (Shanghai, China). Uninoculated spinach leaves and strains subjected to the same Cd stress served as control setups. Total RNA was extracted from the Cd-stressed spinach leaves, with concentration and purity assessed using the Nanodrop 2000 instrument, and integrity evaluated via agarose gel electrophoresis. The RNA Quality Number (RQN) was determined using the Agilent 5,300 system. mRNA was isolated from total RNA using magnetic beads and Oligo(dT) based on A-T base pairing. The mRNA was fragmented into approximately 300 bp fragments using a fragmentation buffer. Single-stranded cDNA was synthesized from mRNA using random primers and reverse transcriptase, followed by the synthesis of double-stranded cDNA. End repair generated blunt ends, an adenine base was added to the 3′ end, and adapter sequences were ligated. Adapter-ligated products were purified, size-selected, PCR amplified, and the final sequencing library was obtained for sequencing after purification. Clean reads from the samples were aligned to the reference genome sequences of *Spinacia oleracea* (version: GCF_020520425.1, https://www.ncbi.nlm.nih.gov/datasets/genome/GCF_020520425.1/) and *E. acetylicum* (version: GCF_018604565.1, https://www.ncbi.nlm.nih.gov/datasets/genome/GCF_018604565.1/) using TopHat2 software for sequence alignment analysis. Genes with significantly different expression levels were identified based on transcript abundance (measured in fragments per kilobase per million reads, FPKM) using a significance test with combined thresholds (FDR ≤ 0.01 and fold change ≥ 2). The reliability of the transcriptome was assessed using Pearson’s correlation coefficient, with each sample having three biological replicates. Differential gene expression analysis was conducted using the Majorbio cloud computing platform, which utilized a series of DESeq software packages.

### 2.11 Statistical analysis and data visualization

Statistical analyses were conducted utilizing SPSS Statistics software (version 17.0) and Microsoft Excel (version 2022), employing independent *t*-tests (*p* < 0.05) or one-way ANOVA as appropriate. Statistical analyses and graphical representations were generated using GraphPad Prism 10 software. Data visualization was executed with the R programming language.^4^ The abstract image was designed using FigDraw.^5^

## 3 Results

### 3.1 The physiological characteristics and Cd-removal property of *Exiguobacterium acetylicum* 4-3-1

**Figure 1:**
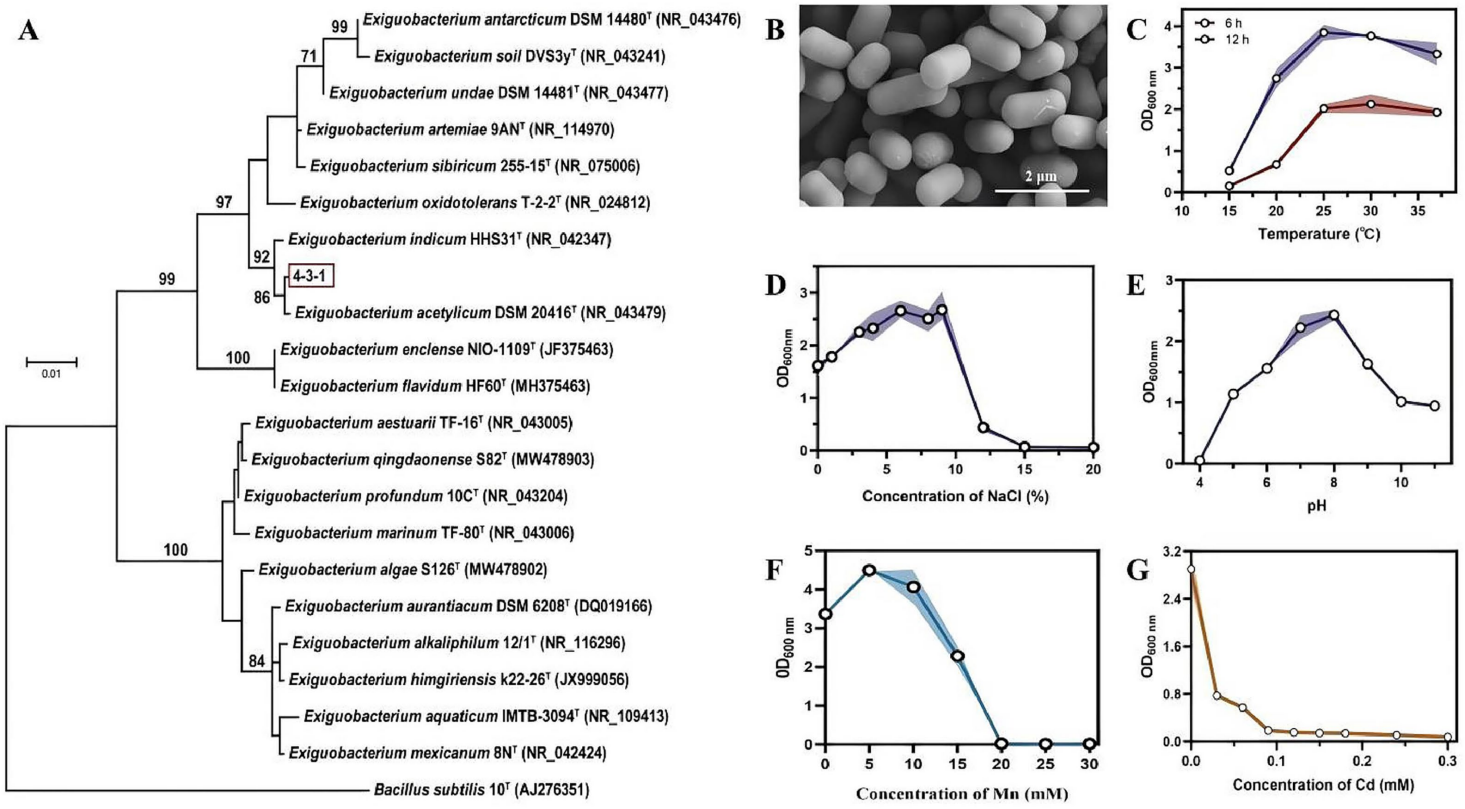
Growth characteristics and functional characterization analysis of *E. acetylicum* 4-3-1. **(A)** Phylogenetic analysis of *E. acetylicum* 4-3-1; **(B)** scanning electron microscopy observation of *E. acetylicum* 4-3-1; **(C–G)** represent the optimal growth temperature, optimal growth NaCl concentration, and optimal growth pH value of *E. acetylicum* 4-3-1, respectively; **(E)** growth of the strain 4-3-1 under different MnCl_2_ concentrations; **(F)** growth of the strain 4-3-1 under different CdCl_2_ concentrations; **(C–G)** represent the mean values from three biological replicates (*n* = 3). Analysis was conducted using the *t*-test method. Compared to the control group, **** indicates a highly significant difference (*p* < 0.0001).

Strain 4-3-1 was isolated from the rhizosphere soil of *Suaeda salsa*. Analysis using the BLAST and EzBioCloud databases revealed that its 16S rRNA gene sequence shares an 99.93% similarity with *E. acetylicum* TC1-3. Phylogenetic tree analysis further indicated that this strain clusters within the same branch as *E. acetylicum*, and thus it was designated as *E. acetylicum* 4-3-1 (Figure 1A).

Based on the morphological characteristics observed through scanning electron microscopy (SEM), the *E. acetylicum* 4-3-1 cells appear plump and exhibit a typical short rod-shaped morphology. The ends of the cells are blunt and rounded, with no observable bending or branching (Figure 1B). Through the investigation of the optimal growth properties of *E. acetylicum* 4-3-1, it was determined that the optimal temperature for growth is 25 °C, the ideal NaCl concentration is 5.0%, which suggests that it is a moderately halophilic bacterium. Furthermore, the optimal pH level was 8.0, indicating a greater tolerance to alkaline environments (Figures 1C–E). Strain 4-3-1 demonstrates significant tolerance to heavy metals. Using an OD_600 nm_ threshold of less than 0.1 as the criterion for complete inhibition, the minimum inhibitory concentration (MIC) of *E. acetylicum* 4-3-1 for MnCl_2_ was determined to be 20 mM, while the MIC for CdCl_2_ was found to be 0.3 mM (Figures 1F,G).

### 3.2 *Exiguobacterium acetylicum* 4-3-1 promoted the growth of spinach

**Figure 2:**
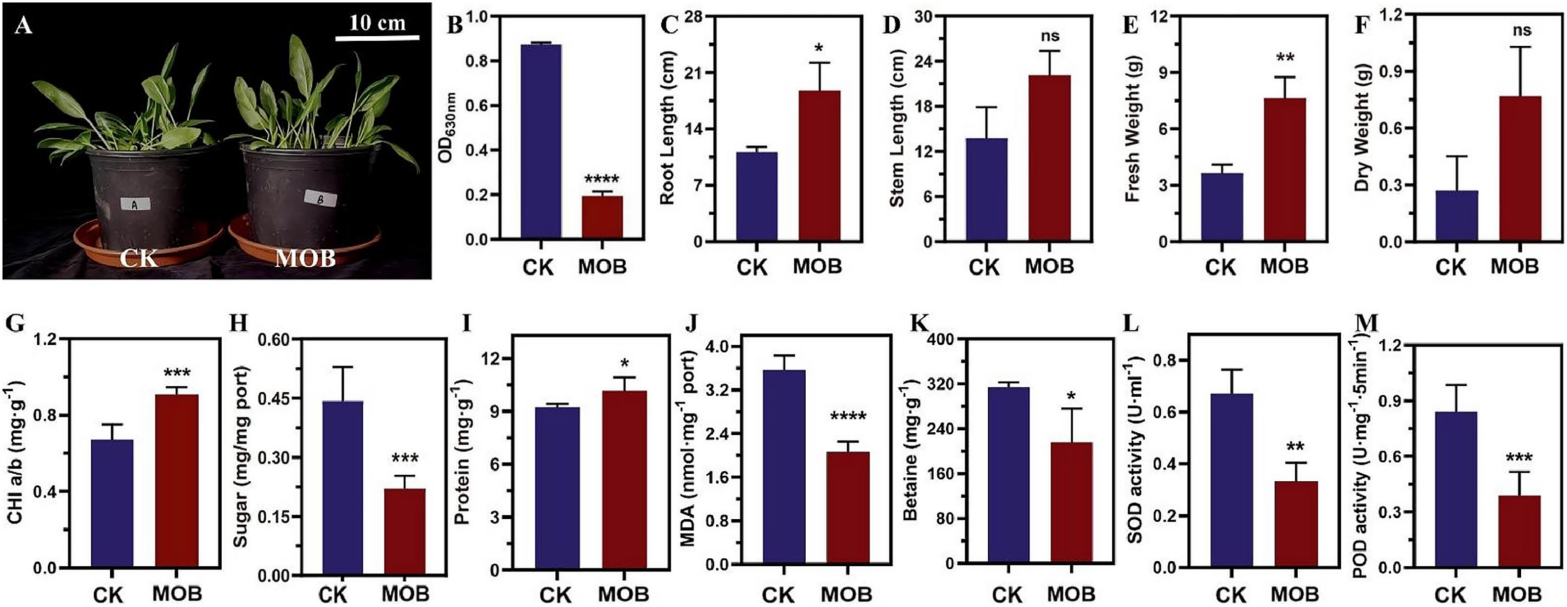
Comparison of growth phenotypic characteristics and physiological and biochemical changes between spinach inoculated with *E. acetylicum* 4-3-1 (MOB) and uninoculated control group (CK). **(A)** Phenotypic observation of spinach in MOB and CK groups; **(B)** Siderophore production ability of *E. acetylicum* 4-3-1. **(C–M)** Comparison of root length, stem length, fresh weight, dry weight, chlorophyll content, soluble sugar, soluble protein, malondialdehyde (MDA), betaine, superoxide dismutase (SOD), and peroxidase (POD) content in spinach plants with and without *E. acetylicum* 4-3-1 inoculation. Data in figures **(B–M)** represent the average of three biological replicates. Analysis was conducted using the *t*-test method. Compared to the control group, **** indicates a highly significant difference (*p* < 0.0001), *** indicates a relatively significant difference (*p* < 0.001), ** indicates a significant difference (*p* < 0.01), * indicates a difference (*p* < 0.05), and ns indicates no significance.

The quantitative detection of siderophore content using the Chrome Azurol S (CAS) assay revealed that *E. acetylicum* 4-3-1 exhibited a significantly higher siderophore production (AS/AR = 0.195/0.874, *p* < 0.0001) (Figure 2B). Meanwhile, the quantitative determination of indole acetic acid (IAA) production using the PC colorimetric assay demonstrated that *E. acetylicum* 4-3-1 could produce 6 mg/L of IAA after 7 days of cultivation, indicating the potential of *E. acetylicum* 4-3-1 to promote plant growth.

To further evaluate the growth-promoting ability of *E. acetylicum* 4-3-1, we co-cultivated this specific strain with spinach under normal soil cultivation conditions for 25 days. Endophytic bacteria were then isolated from the leaves. Notably, bacterial isolates with 16S rRNA gene sequences exhibiting >99% similarity to that of the inoculated *E. acetylicum* 4-3-1 (and thus classified as the same species) were consistently obtained from the leaves of inoculated plants. In contrast, no such *E. acetylicum* isolates were recovered from the uninoculated control plants. This exclusive recovery from the treatment group, coupled with the sequence identity at the species level, strongly indicates that the inoculated strain 4-3-1, or a closely related variant, successfully colonized the spinach leaves endophytically.

Furthermore, the physiological phenotypic characteristics, along with the physiological and biochemical changes in both groups of spinach, were measured to assess the impact of *E. acetylicum* 4-3-1 on spinach growth (Figure 2A). Spinach inoculated with *E. acetylicum* 4-3-1 (MOB) demonstrated a remarkable 69.4% increase in root length compared to the control group (CK) (Figure 2C). Additionally, there was a 68.56% increase in stem length (Figure 2D), along with increases of 108.84% in fresh weight and 184.34% in dry weight (Figures 2E,F). These results demonstrate that *E. acetylicum* 4-3-1 significantly enhances plant growth.

In terms of physiological and biochemical indicators of the 4-3-1 inoculated group the control group of spinach, the chlorophyll content in spinach leaves treated with *E. acetylicum* 4-3-1 increased by 35.12% compared to the control group (Figure 2G), indicating that the addition of *E. acetylicum* 4-3-1 enhanced photosynthesis and improves the photosynthetic capacity of spinach. Soluble sugars, malondialdehyde (MDA), and betaine, which serve as osmotic adjustment substances in plants, reflect the stress levels experienced by the plants (Gowtham et al., 2016; Neshat et al., 2022; Sandhya et al., 2010; Sperdouli and Moustakas, 2012). Notably, the contents of soluble sugars, MDA, and betaine in the 4-3-1 inoculated group spinach decreased by 50.11, 41.98, and 31.18%, respectively, compared to the control group (Figures 2H,J,K). This suggests that the addition of *E. acetylicum* 4-3-1 optimizes the growth environment for spinach plants, resulting in a corresponding decrease in the levels of osmotic adjustment substances. In this study, the soluble protein content in 4-3-1 inoculated group spinach increased by 10.21% compared to the control group (*p* = 0.0209), indicating enhanced nutritional content of plants (Figure 2I) (Nawaz et al., 2020). The contents of superoxide dismutase (SOD) and peroxidase (POD) are indicators of reactive oxygen species (ROS) levels in plants (Nozik-Grayck et al., 2005; Jiménez et al., 2021). The SOD content in spinach leaves treated with *E. acetylicum* 4-3-1 decreased by 50.58% compared to the control group (Figure 2L), while the POD content decreased by 53.52% (Figure 2M). This indicates that *E. acetylicum* 4-3-1 decreased the ROS levels inside the spinach plants, leading to a corresponding reduction in the levels of these two ROS enzymes.

In summary, *E. acetylicum* 4-3-1 can effectively infect spinach leaves and demonstrates a positive effect on spinach growth and nutritional conditions, highlighting its potential application value in enhancing spinach yield.

### 3.3 *Exiguobacterium acetylicum* 4-3-1 inoculation reduced Cd accumulation in spinach

**Figure 3:**
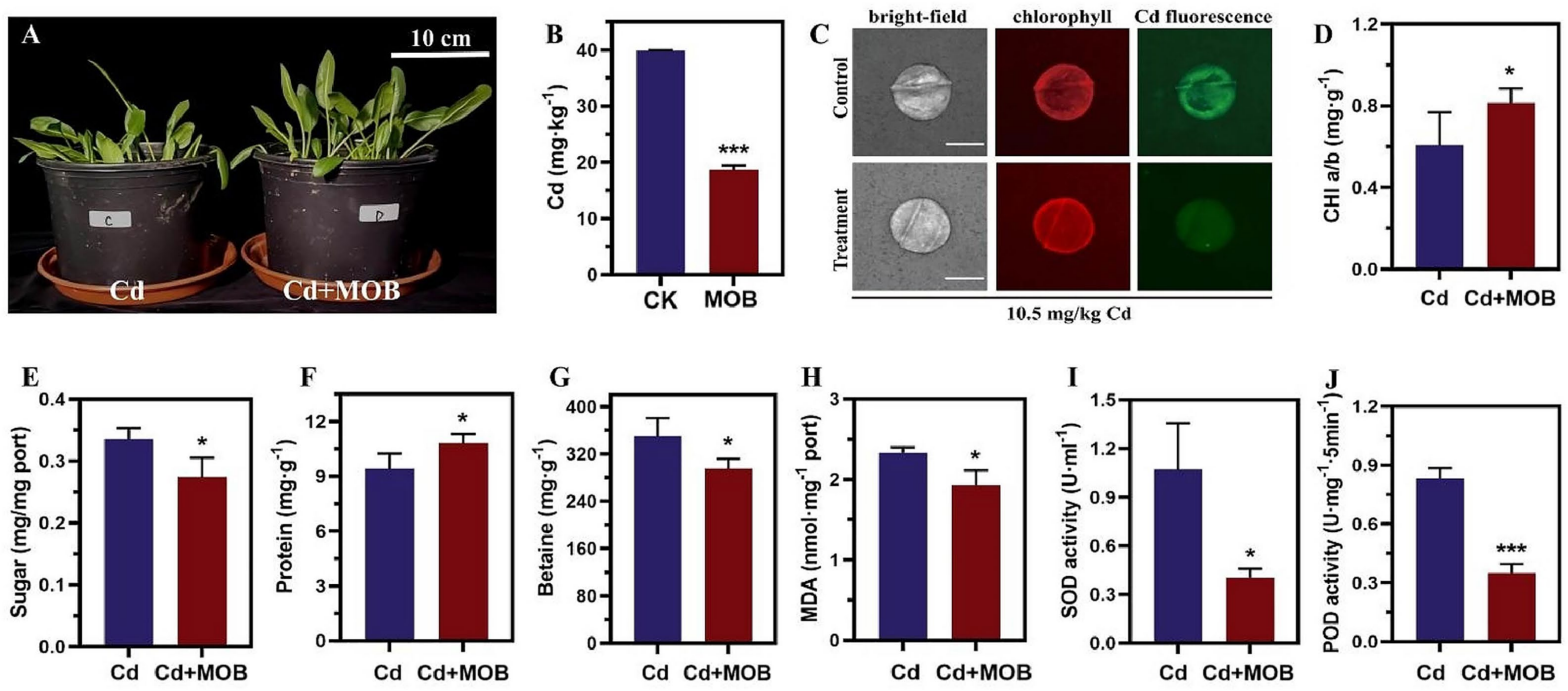
Comparison of physiological and biochemical changes between the *E. acetylicum* 4-3-1 inoculated group (Cd + MOB) and the uninoculated group (Cd) of spinach under Cd stress conditions. **(A)** Phenotypic observation of spinach; **(B)** Cd content in spinach leaves analyzed by ICP-OES, CK: the control with 10.5 mg/kg Cd; MOB: 10.5 mg/kg Cd and with *E. acetylicum* 4-3-1 at OD_600 nm_ = 1; **(C)** Cd fluorescence signals were detected using the Cd-sensitive fluorescent probe (Leadmium™ Green, 488 nm) in the spinach leaves after 25 days of 10.5 mg/kg CdCl_2_ treatment. The chlorophyll signals (561 nm) of the spinach leaves were also detected as a control. Treatment, spinach leaves inoculated with *E. acetylicum* 4-3-1; Control, non-inoculated spinach leaves; **(D–J)** Comparison of chlorophyll content, soluble sugar, soluble protein, betaine, malondialdehyde (MDA), superoxide dismutase (SOD), and peroxidase (POD) levels in spinach plants under Cd stress, with and without *E. acetylicum* 4-3-1 inoculation. Data in figures **(B–J)** represent the average of three biological replicates. Analysis was conducted using the *t*-test method. Compared to the control group, *** indicates a highly significant difference (*p* < 0.001), ** indicates a relatively significant difference (*p* < 0.01), * indicates a significant difference (*p* < 0.05), and ns indicates no significance.

*Exiguobacterium acetylicum* 4-3-1 was co-cultured with spinach for 25 days under 10.5 mg/Kg Cd stress, and its effects of *E. acetylicum* 4-3-1 on spinach plants were evaluated through a series of physiological and biochemical changes. ICP-OES analysis showed that inoculation with *E. acetylicum* 4-3-1 reduced Cd content in Cd-stressed spinach leaves by 53.07% compared to the uninoculated group (Figures 3A,B). This finding indicates that *E. acetylicum* 4-3-1 can effectively mitigate Cd accumulation in spinach leaves. Consistently, a significant reduction in Cd fluorescence signals was detected by the Cd-sensitive fluorescent probe (Leadmium™ Green) in the spinach leaves inoculated with *E. acetylicum* 4-3-1 compared to the uninoculated group (Figure 3C), further confirming that this strain effectively mitigates the Cd accumulation in spinach.

Furthermore, the levels of soluble sugar, MDA, and betaine in the inoculated group under Cd stress decreased by 18.13, 17.14, and 15.79%, respectively, when compared to the Cd group (Figures 3E,G,H). Additionally, the activities of SOD and POD in the inoculated group under Cd stress were reduced by 62.26 and 57.93%, respectively, relative to the Cd group (Figures 3I,J). Meanwhile, the soluble protein content and chlorophyll content in spinach from the inoculated group under Cd stress significantly increased compared to the Cd group, with soluble protein content rising by 14.86% and chlorophyll content increasing by 33.99% (Figures 3D,F).

These results demonstrate that under Cd stress conditions, inoculation with *E. acetylicum* 4-3-1 enhances spinach growth, increases nutrient content, and mitigates oxidative stress, thereby enabling the plants to better withstand the adverse effects induced by Cd stress.

### 3.4 The manganese-oxidizing property of *Exiguobacterium acetylicum* 4-3-1

*E. acetylicum* 4-3-1 was able to tolerate MnCl_2_ with a high concentration of 15 mM (Figure 1F). The manganese-oxidizing activity of the strain was further determined. The presence of BioMnOx, both intracellularly and extracellularly, in *E. acetylicum* 4-3-1 was detected using the LBB method, with a blank culture medium serving as the control. Colorimetric analysis revealed a blue coloration both intracellularly and extracellularly, thereby confirming that the strain 4-3-1 is capable of oxidizing Mn(II) to higher-valent manganese oxides (Supplementary Figure S1).

**Figure 4:**
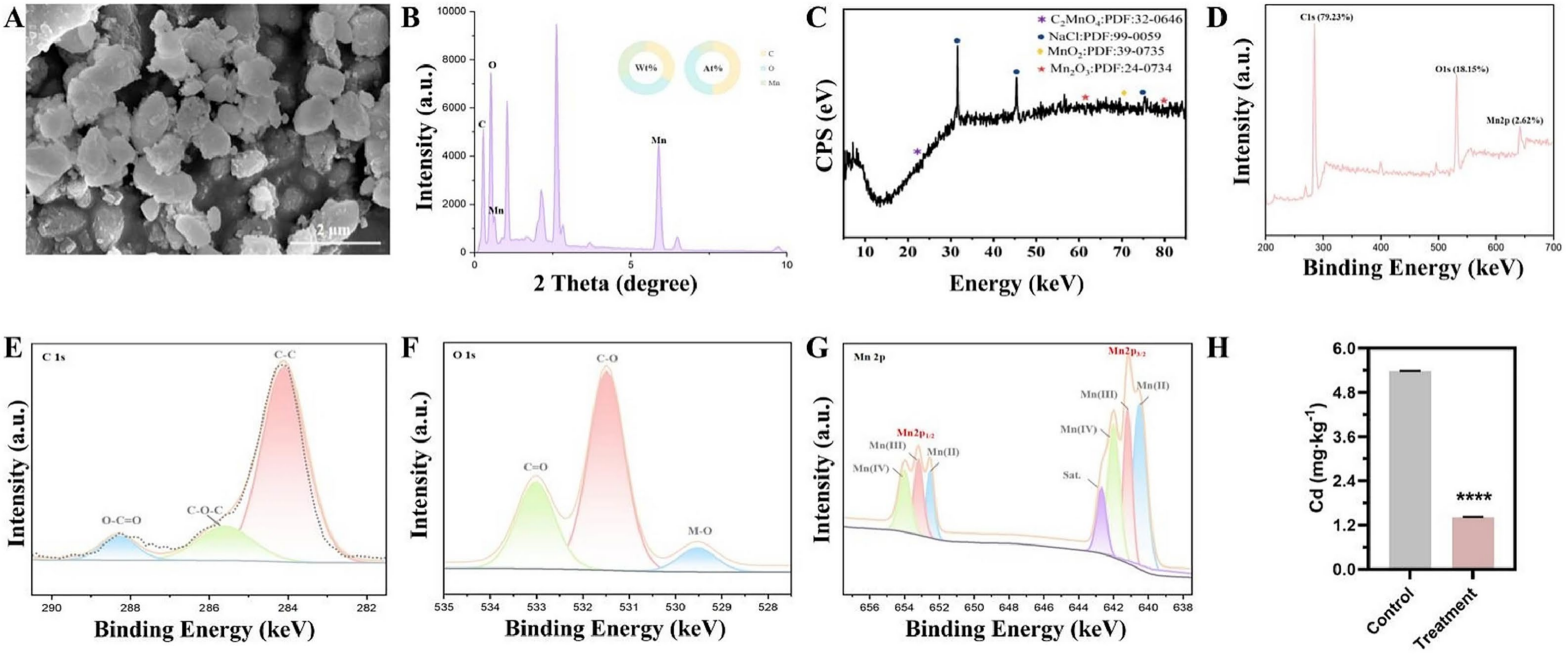
Comprehensive analysis of manganese oxidation activity and structural characteristics of Mn oxidation products in *E. acetylicum* 4-3-1. **(A)** Scanning electron micrograph of Mn oxidation product aggregates in *E. acetylicum* 4-3-1, scale bar, 2 μm; **(B)** Content of carbon (C), oxygen (O), and Mn elements in Mn oxidation product aggregates in *E. acetylicum* 4-3-1; **(C)** XRD pattern of Mn oxidation products in *E. acetylicum* 4-3-1; **(D–G)** Full spectrum and detailed spectra of C, O, and Mn from XPS analysis of Mn oxidation products in *E. acetylicum* 4-3-1; **(H)** Removal of free cadmium (Cd) content from solution by *E. acetylicum* 4-3-1. The data presented in panels.

To further investigate the morphology and elemental composition of the BioMnOx of *E. acetylicum* 4-3-1, we utilized SEM in conjunction with EDS analysis. This BioMnOx exhibits an irregular spherical morphology. EDS analysis identifies three elements on the surface of the irregular spherical structures: carbon (C) at 33.26% weight% (wt) and 49.96% atomic% (at), oxygen (O) at 35.18% wt and 39.67% at, and Mn at 31.56% wt and 10.36% at (Figures 4A,B).

The phase composition of the BioMnOx in *E. acetylicum* 4-3-1 was further characterized by XRD. Figure 4C illustrates that the BioMnOx display a prominent peak at the angle (2θ = 22.4°), which corresponds to manganese oxalate (C_2_MnO_4_) as identified in JCPDS 32-0646. Additionally, the peak observed at 70.9° indicates the presence of manganese dioxide (MnO_2_), referenced in JCPDS 39-0735. Furthermore, the peaks at 61.7° and 79.6° imply the existence of Mn_2_O_3_ with a spinel-like structure, as denoted by JCPDS 24-0734 (Figure 4C). These results indicate that the BioMnOx catalyzed by *E. acetylicum* 4-3-1 possesses more than one oxidation state.

Further investigation into the surface electronic structure and chemical state of BioMnOx catalyzed by *E. acetylicum* 4-3-1 was conducted using XPS. The C1s spectrum of BioMnOx primarily exhibits peaks corresponding to C-C (284.6 eV), C-O-C (285.8 eV), and O-C=O (288.3 eV) (Figure 4D). After correcting for the charge based on the C1s fine spectrum, the O1s spectrum (Figure 4F) can be deconvoluted into lattice oxygen (529.5 eV) and organic oxygen-containing functional groups (531.4 eV and 532.9 eV). This indicates the presence of significant oxidation state characteristics on the surface of the BioMnOx. The Mn2p spin-orbit splitting produces two main peaks: Mn2p 3/2 and Mn2p 1/2, with the former exhibiting four sub-peaks and the latter three. This observation indicates that BioMnOx, catalyzed by *E. acetylicum* 4-3-1, is a mixed-valent manganese compound. The peaks at 652.5 eV (Mn2p 1/2) and 640.2 eV (Mn2p 3/2) correspond to C_2_Mn^II^O_4_, while those at 653.2 eV and 641.2 eV are associated with Mn^III^_2_O_3_ Additionally, peaks at 653.9 eV and 641.9 eV relate to Mn^IV^O_2_. These results confirm the presence of Mn(II), Mn(III), and Mn(IV) in BioMnOx, with Mn(IV) being the most abundant (Figures 4E,G).

Overall, these data confirm the manganese (Mn(II)) oxidation ability of *E. acetylicum* 4-3-1, which may further transform Cd(II) into non-absorbable forms such as Cd or Cd(IV), thereby blocking its uptake by plants. Additionally, the BioMnOx produced by *E. acetylicum* 4-3-1, characterized by low crystallinity and high surface area, may effectively adsorb or precipitate Cd(II) ions. Consistently, under conditions of 0.05 mM CdCl_2_, strain 4-3-1 can effectively reduce the concentration of free CdCl_2_ by 73.74% (Figure 4H). Therefore, the manganese-oxidizing ability of *E. acetylicum* 4-3-1 allows immobilizing free Cd(II) and reduces its concentration in the rhizosphere, consequently minimizing its translocation into plant tissues.

### 3.5 Co-inoculation significantly influenced gene expression in both spinach and strain 4-3-1 under Cd stress

**Figure 5:**
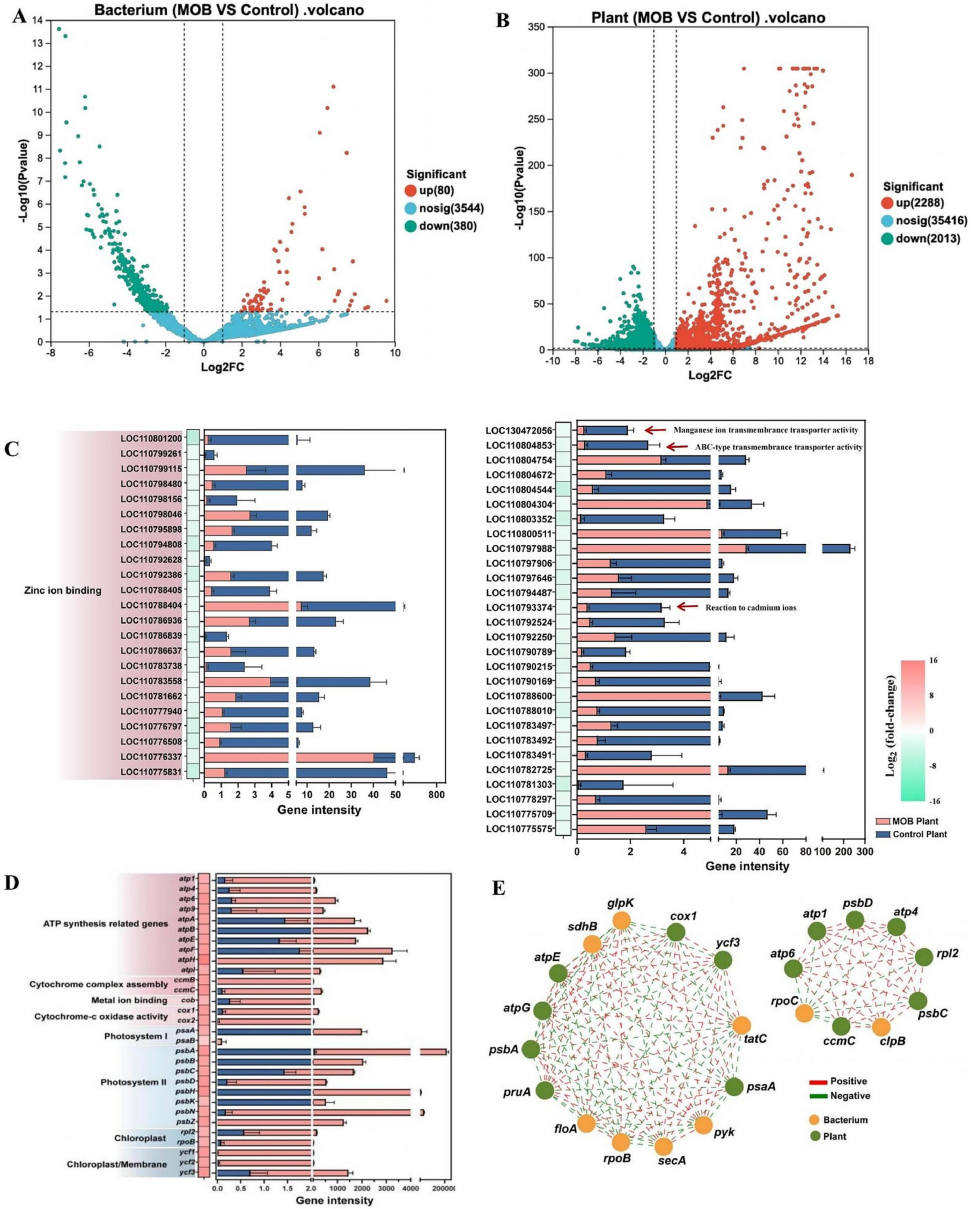
Study on gene expression and related functions of spinach and *E. acetylicum* 4-3-1 under Cd stress. **(A)** Volcano plot of gene expression differences in *E. acetylicum* 4-3-1; **(B)** Volcano plot of gene expression differences in spinach; **(C)** Partial down-regulated genes associated with Cd stress in spinach, |Log_2_ (fold-change)| ≥ 1; **(D)** Partial up-regulated genes associated with Cd stress in spinach, |Log2 (fold-change)| ≥ 1; **(E)** Correlation of cross-species differentially expressed genes between bacteria and spinach. Data in **(A–E)** are the average values of three biological replicates.

To better understand the interactive relationships between *E. acetylicum* 4-3-1 and spinach, a dual transcriptomic analysis was conducted using an RNA-seq approach. The analysis of gene expression levels based on the Pearson correlation coefficient revealed strong correlations among the three biological replicates of spinach samples and those of *E. acetylicum* 4-3-1 (Supplementary Figure S2). In the co-culture system, the spinach plants exhibited 2,288 up-regulated genes and 2,013 down-regulated genes compared with the uninoculated plant controls. In contrast, strain 4-3-1 displayed 80 up-regulated genes and 380 down-regulated genes when compared to the non-co-culture strain (Figures 5A,B). The GO enrichment analysis (Supplementary Figure S3) revealed elevated transcriptional levels of functional modules associated with energy metabolism, including the photosynthesis module, nucleoside triphosphate biosynthetic process module, ATP biosynthetic process module, and ATP metabolic process module. Consistently, KEGG analysis (Supplementary Figure S5) demonstrated an increased transcription of genes associated with nine metabolic pathways, including photosynthesis, oxidative phosphorylation, and plant hormone signal transduction. This suggests that *E. acetylicum* 4-3-1 may enhance the adaptation of spinach to adverse conditions by improving its energy metabolism. Specifically, under Cd stress, spinach inoculated with *E. acetylicum* 4-3-1 showed significant gene up-regulation: *atpH* increased over 15-fold (in log_2_fold-change), *atpE* and *atpF* by 12-fold, the cytochrome C encoding genes *cox1* by 12-fold, and *cox2* by 11-fold. Photosystem-related genes *psbH* and *psbA* were upregulated by 13.5-fold and nearly 13-fold, respectively. Notably, the results demonstrated a significant upregulation of the ycf1, ycf2, and ycf3 genes, with expression levels increasing by 12.7-fold, 11.7-fold, and 12.6-fold, respectively (Figure 5D). These *ycf* genes are associated with resistance to heavy metals, particularly lead and Cd (Song et al., 2003). These changes suggest that *E. acetylicum* 4-3-1 enhances the response of spinach to Cd stress and aids in detoxification.

Moreover, a reduction was observed in the transcriptional abundance of genes associated with transport processes in spinach, specifically those involved in zinc and Mn ion transport (Supplementary Figure S4). The transcriptional levels of genes related to zinc ion binding were significantly down-regulated, with fold changes ranging from 1 to 4 (Figure 5C). Importantly, zinc and Mn transporters are the primary pathways for Cd entry into plants (Hussain et al., 2004; Wong and Cobbett, 2009). Therefore, the incubation of *E. acetylicum* 4-3-1 may decrease Cd absorption and translocation in spinach, thereby mitigating stress and enhancing growth conditions.

Based on the preceding analysis, a differential expression correlation study was performed to examine the relationship between differentially expressed genes associated with Cd stress in the plant species spinach and those differentially expressed genes with an adjusted *p*-value of less than 0.05 in the 4-3-1 group (Figure 5E). According to the Spearman correlation coefficient, only nodes ranked within the top 25 in terms of abundance and exhibiting correlation coefficients of |r| ≥ 0.05 are displayed. In *E. acetylicum* 4-3-1, the enzyme glycerol kinase (*glpK*), which plays a pivotal role in regulating glycerol uptake and metabolism, was found to be co-expressed with photosystem-related genes (*psbA*, *psaA*) in spinach. In spinach, genes associated with the photosystems, including *psbD* and *psbC*, along with genes implicated in ATP synthesis (*atp1* and *atp6*), exhibit a negative correlation with the *rpoC* gene in *E. acetylicum* 4-3-1. Notably, mutations in the *rpoC* gene have been associated with enhanced production of acetate and amino acids, including proline, as well as the preservation of membrane integrity under stress conditions (Guo et al., 2017). These data suggest that the carbon and nitrogen metabolisms in *E. acetylicum* 4-3-1 are closely related to the photosynthesis of spinach, potentially enhancing the adaptation and survival of spinach under Cd stress conditions.

### 3.6 *Exiguobacterium acetylicum* 4-3-1 inoculation altered altered the rhizosphere microbial composition of spinach under Cd stress

**Figure 6:**
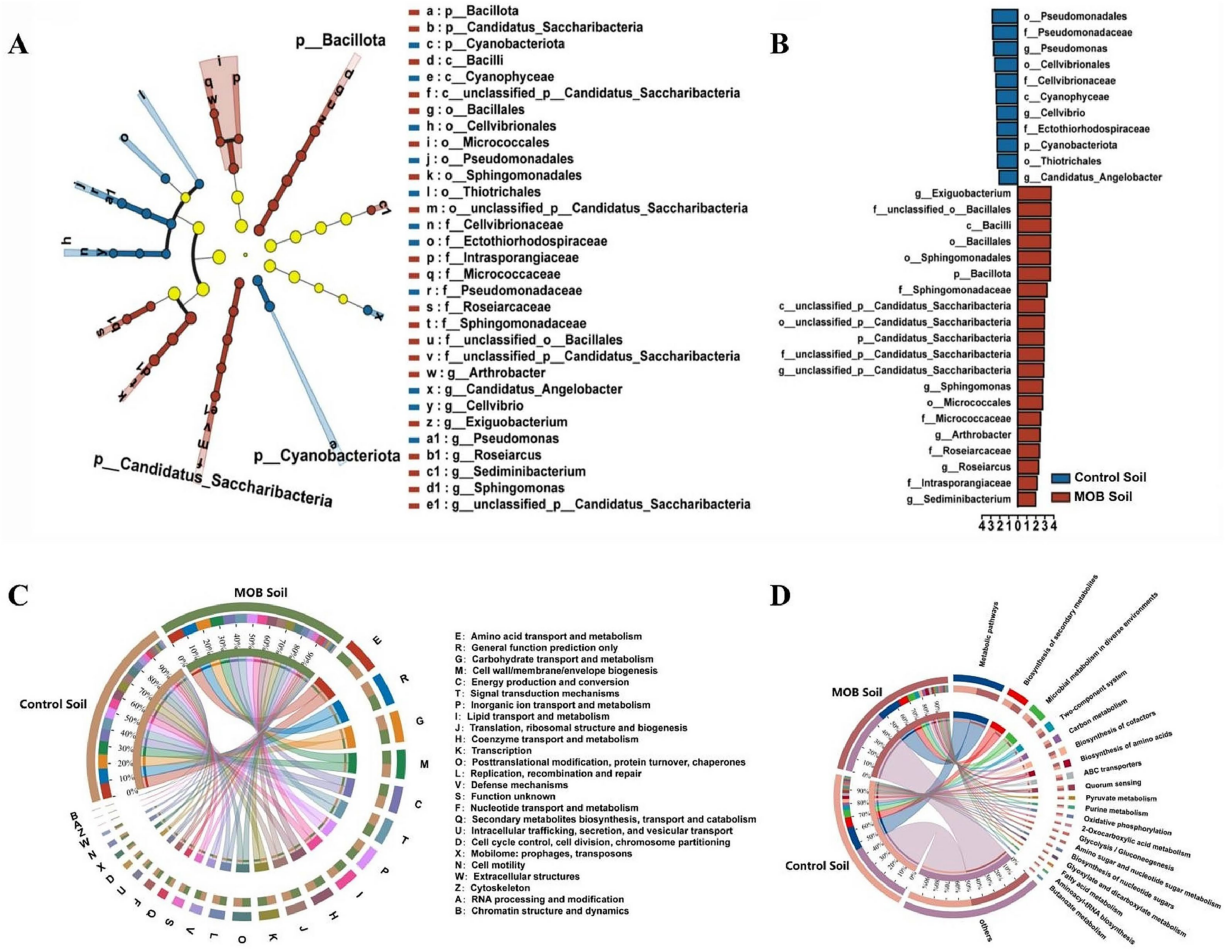
Analysis of soil microbial community abundance and sample differences between the Cd stress control group and the Cd stress group treated with *E. acetylicum* 4-3-1. **(A)** LEfSe species hierarchy diagram. The one-against-all comparison strategy is applied, utilizing the PPM abundance calculation method with LDA > 2; **(B)** LDA discriminant results; **(C)** Functional composition and differences based on the COG database; **(D)** Functional composition and differences based on the KEGG database. Data in figures **(A–D)** represent the average of three biological replicates.

The rhizosphere serves as the primary site for plant-microorganism interactions. In this study, we compared the taxonomic features of rhizosphere microbes associated with spinach using metagenomic sequencing in both the *E. acetylicum* 4-3-1 inoculated group and the uninoculated group of spinach under Cd stress conditions (Supplementary Figure S6). Results showed that following the application of *E. acetylicum* 4-3-1, the abundance of 14 genera increased, while that of 7 genera decreased. Among the 14 genera, *Exiguobacterium* exhibited the most significant increase at 0.94%, followed by *Bradyrhizobium* at 0.53%. Additionally, the abundance of *Pseudolabrys* increased by 0.4%. Besides, the relative abundance of *Actinomadura* (0.65%), *Streptomyces* (0.55%), and *Reticulibacter* (0.55%) in rhizosphere soil were significantly decreased under the strain 4-3-1 inoculation. Furthermore, the LEfSe species hierarchy chart identified a total of 31 biomarker taxa that were significantly different between the two groups (Figure 6A *p* < 0.05), with 11 taxa in the uninoculated setup and 20 taxa in the inoculated setup. Among these taxa, the genus *Exiguobacterium* (LDA = 3.72), which was introduced during the experiment, exhibited the highest differential value between the two groups. In the inoculated group, Bacillales, Sphingomonadales, and Micrococcales were prominent, while Pseudomonadales, Cellvibrionales, and Cyanophyceae dominated the uninoculated control group. These findings indicate that *E. acetylicum* 4-3-1 alters the microbial communities associated with spinach under Cd stress (Figure 6B).

Further analysis of rhizosphere microbial communities utilizing the COG database revealed that *E. acetylicum* 4-3-1 significantly influences rhizosphere microbial functions of spinach under Cd stress (Figure 6C). In comparison to the uninoculated setup, treatment with *E. acetylicum* 4-3-1 resulted in increase in an abundance of coenzyme transport and metabolism, followed by cell wall/membrane/envelope biogenesis (carbohydrate transport and metabolism), lipid transport and metabolism, amino acid transport and metabolism, replication, recombination and repair, along with 15 other COG functions. Conversely, nine functions, including transcription, exhibited significant decreases. According to the KEGG database analysis, in the 4-3-1 treated setup, there is an increase in cofactor biosynthesis, purine metabolism, butanoate metabolism, oxidative phosphorylation, and amino acid biosynthesis, while pathways for fatty acid metabolism, glycolysis/gluconeogenesis, starch and sucrose metabolism, pyruvate metabolism, and quorum sensing decreased compared to the control setup (Figure 6D).

Together, *E. acetylicum* 4-3-1 inoculation modified the rhizosphere soil microbial communities and functions in spinach under Cd stress.

### 3.7 Synergistic effects of *Exiguobacterium acetylicum* 4-3-1 and *Bacillus subtilis* on spinach growth under Cd stress

**Figure 7:**
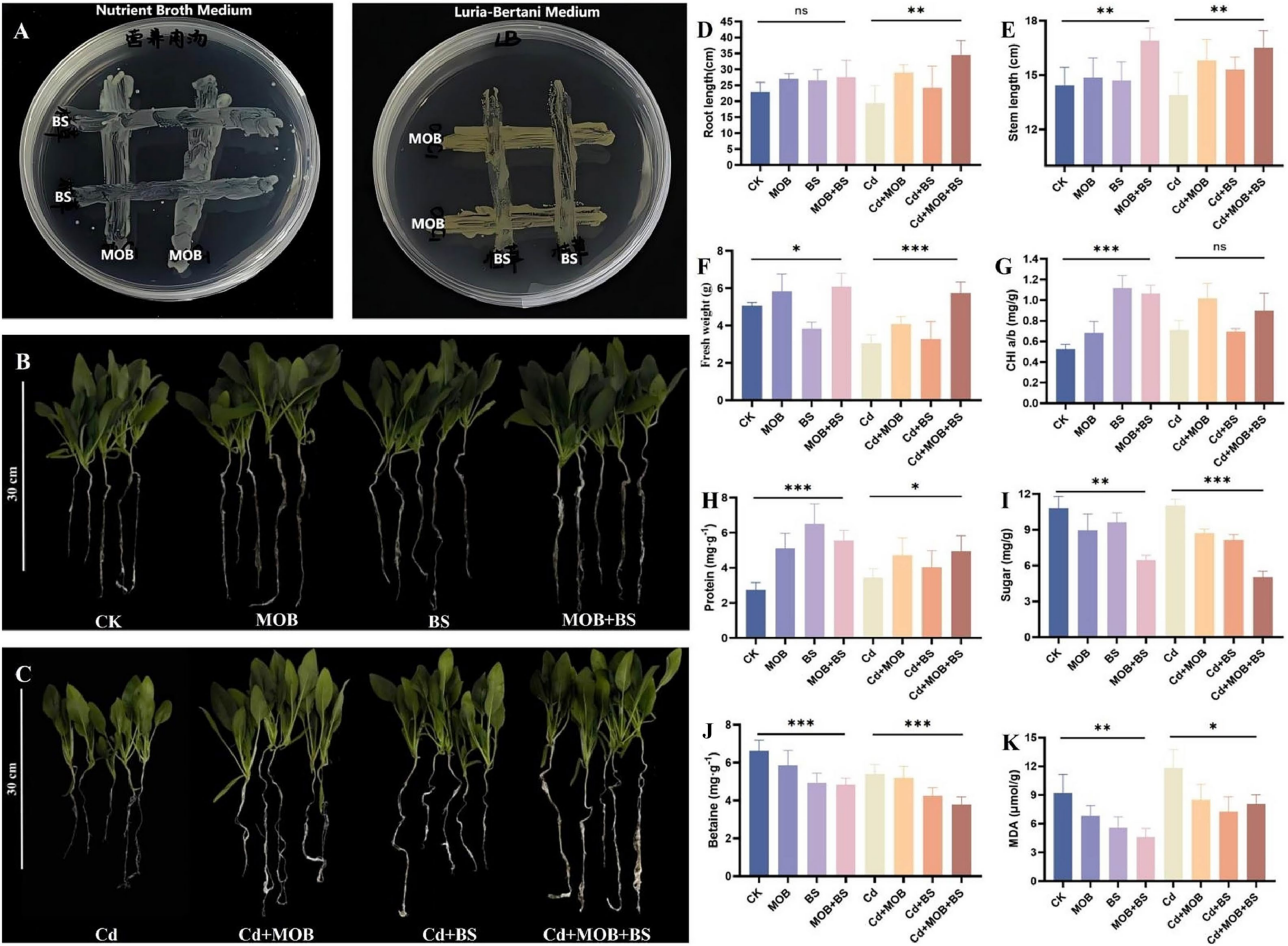
Growth-promoting and detoxification effects of *E. acetylicum* 4-3-1, *Bacillus subtilis* CICC 10155, and synthetic communities on spinach. **(A)** The antagonistic experiment of dual culture between the two strains on two kinds of media. 4-3-1, *E. acetylicum* 4-3-1; Bs, *Bacillus subtilis* CICC 10155; **(B)** Observation of growth phenotypes of four groups of spinach cultivated under normal soil conditions, where CK is the control group, MOB, the group of spinach inoculated with *E. acetylicum* 4-3-1; BS, the group of spinach inoculated with *Bacillus subtilis* CICC 10155; MOB+BS, the group of spinach cultivated with a synthetic community; **(C)** Observation of growth phenotypes in four groups of spinach cultivated under cadmium contaminated soil, where Cd is the control group. Cd + MOB, the spinach group inoculated with *E. acetylicum* 4-3-1; Cd + BS, the spinach group inoculated with *Bacillus subtilis* CICC 10155; Cd + MOB+BS, the spinach group cultivated with a synthetic community; **(D–K)** Comparison of root length, stem length, fresh weight, chlorophyll content, soluble sugar content, soluble protein content, betaine content, and malondialdehyde (MDA) content in spinach among different treatment groups, respectively. Data in figures **(B–K)** represent the average of five biological replicates. Analysis was conducted using the *t*-test method. Compared to the control group, *** indicates a highly significant difference (*p* < 0.001), ** indicates a relatively significant difference (*p* < 0.01), * indicates a significant difference (*p* < 0.05), and ns indicates no significance.

The aforementioned studies have demonstrated that *E. acetylicum* 4-3-1 can enhance the abundance of *Bacillus* species in the rhizosphere of spinach. To further explore the potential growth-promoting and detoxification benefits of this interaction under Cd stress, a synthetic microbial community comprising *E. acetylicum* 4-3-1 and *Bacillus subtilis* CICC 10155 was established and evaluated in spinach (Supplementary Figure S7). The dual culture antagonism assay between two strains revealed no antagonistic effects (Figure 7A). The strain was co-cultured with spinach for 23 days, and the physiological characteristics and biochemical changes of spinach across different treatment groups were assessed (Figures 7B,C). The results indicated that, both in the presence and absence of Cd(II), inoculation with the synthetic community was more effective in promoting spinach growth compared to inoculation with a single strain.

The effect was notably more pronounced in the presence of Cd(II) (Figure 7C). Upon treatment with 10.5 mg/kg CdCl_2_, the root length of spinach treated with the combined microbial strains increased by 19.23% compared to treatment with *E. acetylicum* alone, by 42.74% compared to treatment with *Bacillus subtilis* alone, and by 77.80% compared to the uninoculated control (Figure 7D). Regarding stem length, the synthetic microbial community resulted in a 4.25% increase relative to the group inoculated solely with *E. acetylicum* 4-3-1, a 7.97% increase compared to the group inoculated exclusively with *Bacillus subtilis*, and an 18.85% increase compared to the control group (Figure 7E). The synthetic community also enhanced spinach fresh weight by 40.86% over the *E. acetylicum* 4-3-1 group, by 75.04% over the *Bacillus subtilis* group, and by 89.14% over the control (Figure 7F). In terms of soluble protein content, a key indicator of nutritional value, the synthetic community demonstrated an 8.75% increase compared to spinach inoculated with *E. acetylicum* 4-3-1 alone and a substantial increase of 101.84% compared to the control group (Figure 7H). Furthermore, the synthetic community reduced soluble sugar content by 42.11, 38.11, and 54.16% compared to these respective groups (Figure 7I). Additionally, betaine levels decreased by 27.24, 11.18, and 29.94% compared to the same groups (Figure 7J). Although the MDA content in spinach showed minimal differences between the synthetic community and single-strain cultures, it demonstrated a significant reduction of 31.75% when compared to the control group (Figure 7K).

## 4 Discussion

### 4.1 *Exiguobacterium acetylicum* 4-3-1 is a plant growth-promoting rhizobacterium that reduces Cd uptake in spinach

The bacterial genus *Exiguobacterium* includes various species from diverse environments, with research has focused on their biotechnological applications such as enzyme production, bioremediation, and pollutant degradation (Kasana and Pandey, 2018). Some isolates exhibit plant growth-promoting capabilities and are currently being explored for their potential to enhance agricultural production (Dargiri et al., 2025). In this study, we describe a strain of *E. acetylicum* 4-3-1 that effectively reduces the accumulation of the heavy metal Cd in spinach. Isolated from plant rhizosphere soil, strain 4-3-1 demonstrates the ability to produce indole-3-acetic acid (IAA) and siderophores, crucial for growth and stress responses, alongside a notable tolerance to heavy metals. Co-cultivation of spinach with strain 4-3-1 resulted in a significant enhancement in root development and overall plant growth, highlighting its potential as a plant growth-promoting rhizobacterium. Furthermore, strain 4-3-1 exhibited manganese-oxidizing capabilities, demonstrating the ability to transform Mn(II) into higher oxidation states such as Mn(III) and Mn(IV). Manganese oxides can further modify the rhizosphere environment by accelerating ammonia oxidation and enhancing soil organic matter degradation (Wang et al., 2021; Sunda and Kieber, 1994), thus facilitating carbon and nitrogen cycling, which in turn promotes the growth of plants under nutrient-poor condition.

Strain 4-3-1 can also effectively reduce Cd content in spinach, as confirmed here by ICP-MS and Cd-specific fluorescence probe detection. Cd stress adversely affects photosynthetic efficiency by reducing chlorophyll content and compromising photosystem II (PSII) efficiency (Sunda and Kieber, 1994; Haider et al., 2021). Additionally, it elevates oxidative stress, as indicated by increased levels of malondialdehyde (MDA) and reactive oxygen species (ROS), which can harm cellular membranes and other components (Shahid et al., 2020). Inoculating spinach plants with *E. acetylicum* 4-3-1, however, enhances chlorophyll content and reduces MDA levels under Cd stress, while also decreasing superoxide dismutase and peroxidase activities. This indicates that this strain alleviates oxidative stress and promotes plant growth under Cd stress. Moreover, *E. acetylicum* is a representative Firmicutes bacterium found in the healthy human gut microbiota (Bae et al., 2016). Its unique traits, including plant growth promotion, Cd reduction, and harmless to healthy individual, render it a promising resource for mitigating heavy metal contamination in crops, thus contributing to soil ecological restoration and sustainable agriculture. Spinach, known for Cd tolerance and accumulation, was used in the study. Future research should explore the long-term impact of *E. acetylicum* 4-3-1 on Cd uptake in non-hyperaccumulating crops in native soils, ensuring safety and effectiveness for soil health and crop production.

### 4.2 The endogenous mechanisms of *Exiguobacterium acetylicum* 4-3-1 in reducing Cd accumulation in spinach

Dual transcriptome sequencing analysis revealed the molecular mechanism by which *E. acetylicum* 4-3-1 reduces Cd content inside spinach. Strain 4-3-1 can endophytically penetrate plant tissues, as it was isolated from the leaves of spinach incubated with this strain. The analysis showed a decrease in the transcriptional abundance of genes associated with transport processes, particularly those involved in Zn and Mn ion transport. Notably, a specific transporter for Cd uptake has yet to be characterized. The uptake of Cd by plant roots often involves competition between Cd and essential mineral elements that share similar chemical properties at the absorption sites. Previous studies have confirmed an antagonistic relationship between Zn, Mn, and Cd during their active uptake, indicating that Cd enters plants through Zn or Mn channels (Muehe et al., 2015; Yan et al., 2019). Consequently, the reduction in Cd accumulation in spinach treated with *E. acetylicum* 4-3-1 may be attributed to the down-regulation of Zn/Mn transporter genes.

Soluble antioxidants, such as glutathione, as well as antioxidant enzymes like glutathione reductase (GR) and peroxidases (POD), play crucial roles in metal detoxification by facilitating metal chelation and providing antioxidant protection. Our findings revealed a down-regulation of ROS detoxification-related genes, including glutathione S-transferase and peroxisome biogenesis genes, in spinach treated with *E. acetylicum* 4-3-1, indicating alleviation of heavy metal stress. This observation is consistent with the measured physiological levels of POD content, suggesting that treatment with this strain mitigated the ROS levels induced by heavy metal stress.

Furthermore, the transcription of photosynthesis-related genes *psbA*, *psbC*, and *psbD* was found to be upregulated. Previous studies have shown that Cd stress inhibits the repair of photodamaged PSII (Qian et al., 2009) by suppressing the transcription of the *psbA* gene, which encodes the essential D1 protein. Additionally, the expression of core PSII proteins, including D2 (*psbD*), CP43 (*psbC*), and CP47 (*psbB*), was reduced, negatively affecting PSII assembly (Muhammad et al., 2020). The upregulation of photosynthesis-related genes in spinach by the strain 4-3-1 suggests improved photosynthetic efficiency and energy availability to mitigate the damage induced by Cd stress. Moreover, the upregulation of ATP synthesis-related genes, such as *atp6* and *atpG*, indicates a potential increase in energy levels, which is consistent with the observed growth promotion and increased biomass in spinach under Cd stress. Consistently, studies have demonstrated the role of these genes in alleviating heavy metal stresses in plants by enhancing the expression of photosynthesis and energy metabolism genes (Sen et al., 2014; Sun et al., 2025).

Together, these data provide insights into the endogenous mechanisms by which the strain 4-3-1 reduces Cd accumulation and supports the growth of spinach under Cd stress. This is achieved by enhancing the expression levels of genes associated with photosynthesis and energy metabolism, while simultaneously downregulating the genes encoding transporters responsible for heavy metal uptake.

### 4.3 The exogenous mechanisms of *Exiguobacterium acetylicum* 4-3-1 in the migration of Cd to spinach

As a rhizosphere manganese-oxidizing bacterium, *E. acetylicum* 4-3-1 is proposed to reshape the rhizosphere functions, which can alter the bioavailability and toxicity of Cd, thereby affecting their mobility and uptake in soil. Firstly, this strain enhances the growth of other functional microorganisms, such as Bacillales, Sphingomonadales, and Micrococcales, which possess plant growth-promoting and metal-immobilizing capabilities (Wang et al., 2025; Zhang et al., 2025). For instance, Bacillales exhibits strong surface adsorption (He et al., 2025), accumulating Cd intracellularly (Yao et al., 2021) and mitigating its toxicity by forming insoluble precipitates (Wang et al., 2021; Jabeen et al., 2022). Consistently, in this study, the combination of *E. acetylicum* 4-3-1 and *Bacillus subtilis* showed synergistic effects on spinach growth under Cd stress.

Secondly, *E. acetylicum* 4-3-1 facilitates the transformation of Mn(II) into BioMnOx, which includes Mn(III) and Mn(IV). These BioMnOx are capable of transforming free Cd^2+^ into stable complexes through mechanisms such as electrostatic adsorption, coprecipitation, and redox reactions, thereby significantly reducing its bioavailability (Chen et al., 2023; Liu et al., 2024). Research has demonstrated that the application of MnO_2_ and Mn_2_O_3_ reduces bioavailable Cd in the rice rhizosphere by 28.9 and 15.3%, respectively (Zhang et al., 2025). Moreover, studies have confirmed that manganese-oxidizing bacteria can promote the formation of iron-manganese plaques on plant root surfaces. For instance, the co-cultivation of manganese-oxidizing bacteria *Pantoea eucrina* SS01 and *Pseudomonas composti* SS02 with *Suaeda salsa* facilitates the deposition of manganese oxides on the roots (Zhao et al., 2019). Similarly, in rice, manganese-oxidizing *Burkholderia* sp. D416 and *Pseudomonas putida* 23,483 have been found to enhance the formation of these plaques, leading to a reduction in the uptake and translocation of Cd by rice roots from the soil (Wei et al., 2021; Liu et al., 2010; Li et al., 2023).

Thirdly, *E. acetylicum* 4-3-1 may influence carbon and nitrogen (C/N) metabolism in the rhizosphere, thereby modifying the soil environment. In the spinach setup treated with 4-3-1, we observed an increase in cofactor biosynthesis, purine metabolism, butanoate metabolism, oxidative phosphorylation, and amino acid biosynthesis. Conversely, pathways associated with fatty acid metabolism, glycolysis/gluconeogenesis, starch and sucrose metabolism, and pyruvate metabolism exhibited a decrease compared to the control setup. The altered nutrient dynamics in the rhizosphere can be further illustrated by changes in the rhizosphere microbiome. Notably, there is an increased abundance of copiotrophic bacteria such as Micrococcales and Sphingomonadales, while a reduction in the abundance of oligotrophic bacteria, such as Cyanophyceae, suggests a potential change in the C/N ratio in the rhizosphere. Future analyses will be necessary to identify changes in specific metabolites in the rhizosphere, employing methods such as non-targeted metabolomics.

**Figure 8:**
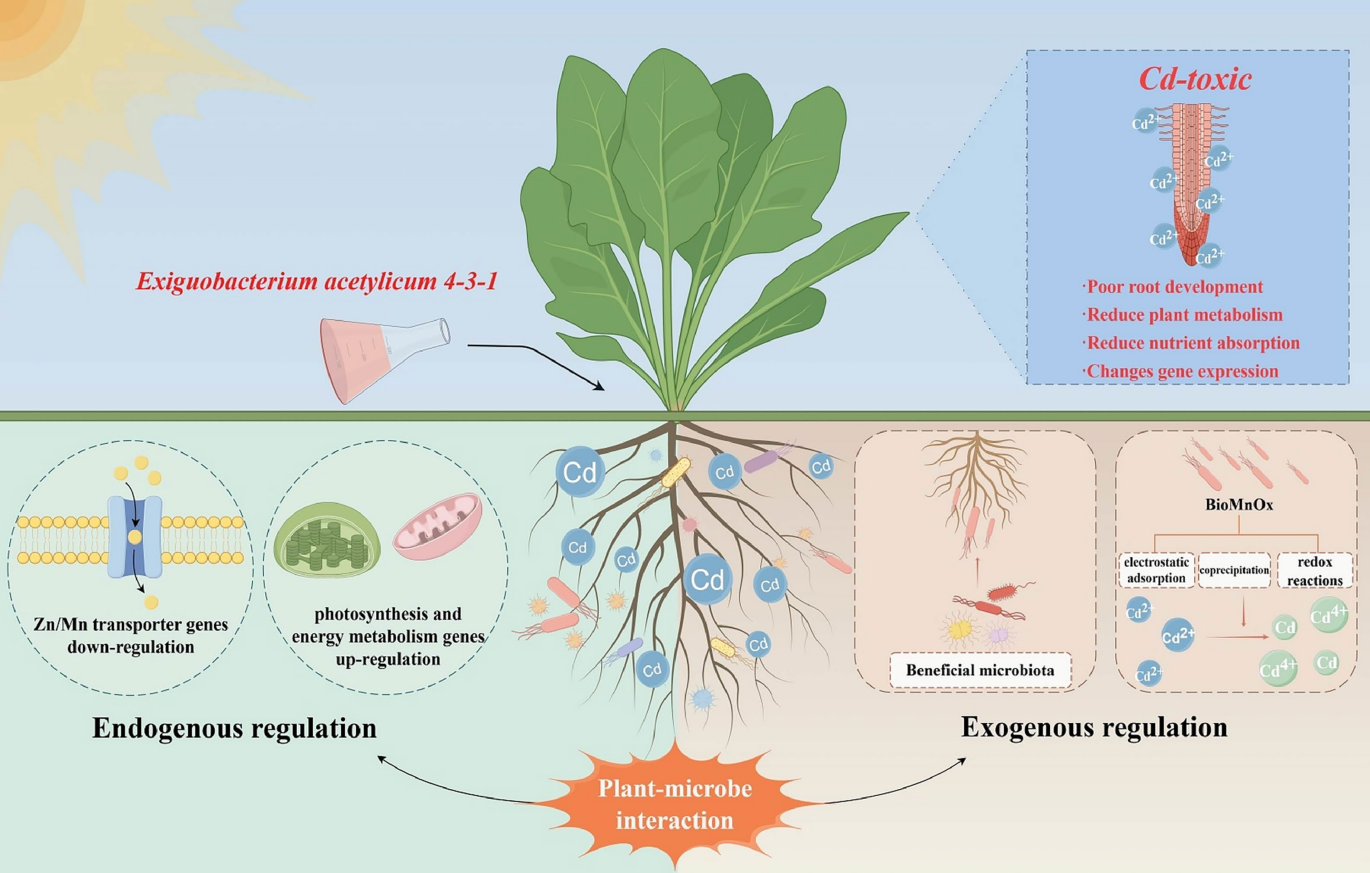
Under Cd stress, the endogenous and exogenous regulatory mechanisms of *E. acetylicum* 4-3-1 for detoxification in spinach.

Overall, *E. acetylicum* 4-3-1 is a potent PGPR that enhances spinach growth and reduces Cd accumulation through a multi-faceted mechanism. This mechanism involves both direct and indirect promotion of plant growth and activity, the alterations to the gene expression levels and rhizosphere microbiome in spinach, and the potential Cd adsorption capacity of biogenic Mn oxides produced by the strain. This discovery provides insights for the targeted manipulation of rhizosphere bacteria to mitigate heavy metal stress. Currently, *E. acetylicum* is intractable to molecular genetic manipulation (Bae et al., 2016); however, with advancements in genetic engineering techniques, it may become feasible to enhance its beneficial traits, thereby increasing its application as a novel, sustainable biofertilizer in agricultural practices (Figure 8).

## Data Availability

The datasets presented in this study can be found in online repositories. The names of the repository/repositories and accession number(s) can be found in the article/Supplementary material.

## Footnotes

1
https://blast.ncbi.nlm.nih.gov/Blast.cgi

2
https://www.ezbiocloud.net/

3
http://www.genome.jp/keeg/

4
https://www.r-project.org/

5
https://www.figdraw.com/
