# Supplementary material for: Manganese-oxidizing Exiguobacterium acetylicum 4-3-1 reduces cadmium accumulation in spinach
Source: Front Microbiol. 2026 Jan 13;16:1734825. doi: 10.3389/fmicb.2025.1734825 (PMC12845336; doi:10.3389/fmicb.2025.1734825)
Supplement: Supplementary file 1 [file Supplementary_file_1.docx]

Supplementary data

**Manganese-oxidizing *Exiguobacterium acetylicum* 4-3-1 Reduces Cadmium Accumulation in Spinach**

Yujia Sun^1#^, Mengyao Ding^1,3#^, Wenjuan Zheng^1#^, Haoran Zhang^1^, Zhenkun Lu^1^, Jian Zhang^2^, Guoyan Zhao^1,4^*

^1^College of Life Science, Shandong Normal University, Jinan 250014, China.

^2^College of Geography and Environment, Shandong Normal University, Jinan 250014, China.

^3^College of Resources and Environmental Sciences, China Agricultural University, Beijing 100193, China.

^4^State Key Laboratory of Biobased Material and Green Papermaking, Qilu University of Technology, Shandong Academy of Sciences, Jinan 250353, China.

#These authors contributed equally: Yujia Sun, Mengyao Ding, Wenjuan Zheng

*Corresponding author:

Guoyan Zhao, E-mail address: zhaoguoyan@sdnu.edu.cn, ORCID: 0000-0003-3200-8800.

This supplementary document contains:

Supplementary Figures 1-8


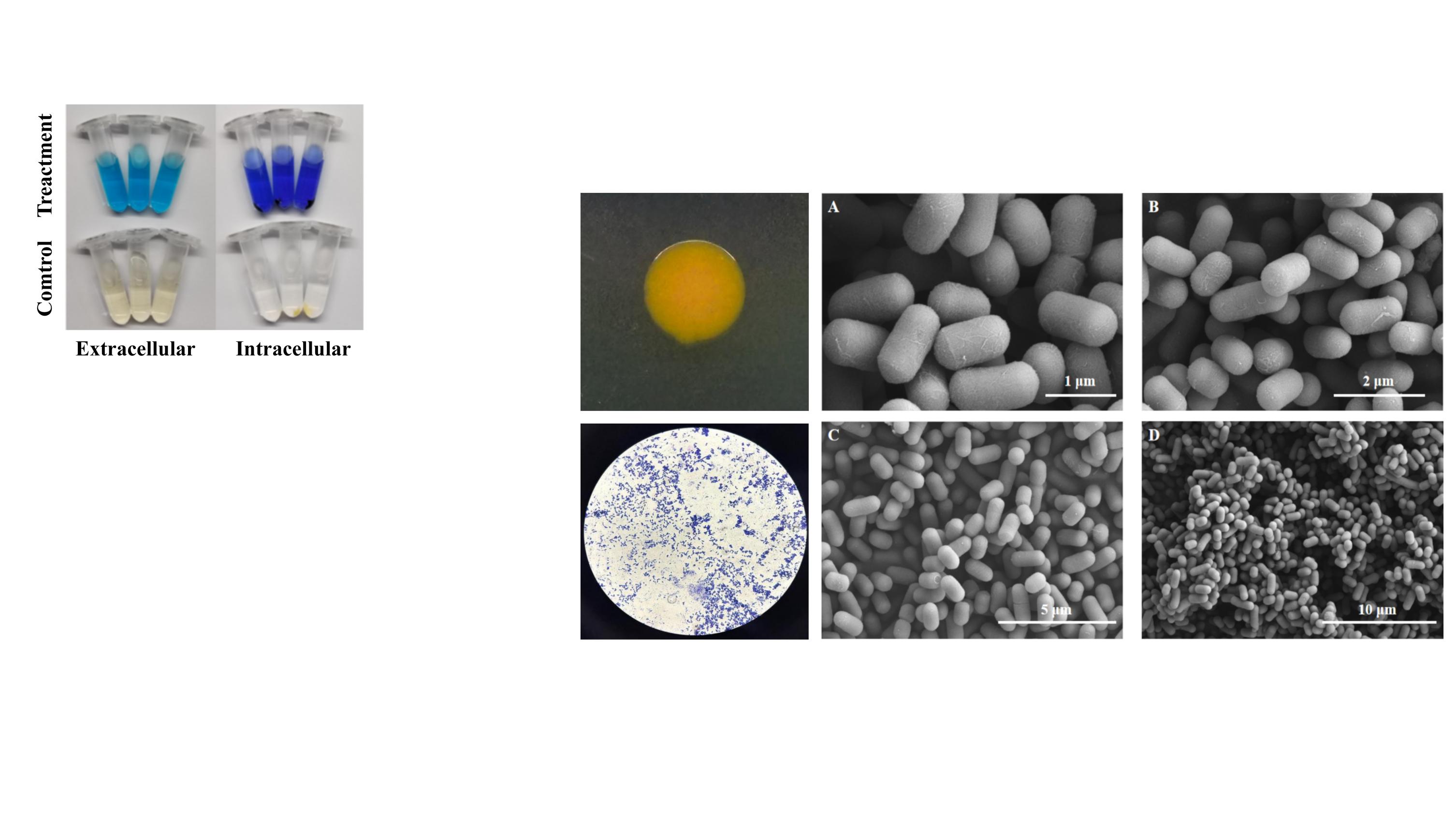


Fig. S1. Comparison of intracellular and extracellular Mn oxidation activity in *E. acetylicum* 4-3-1


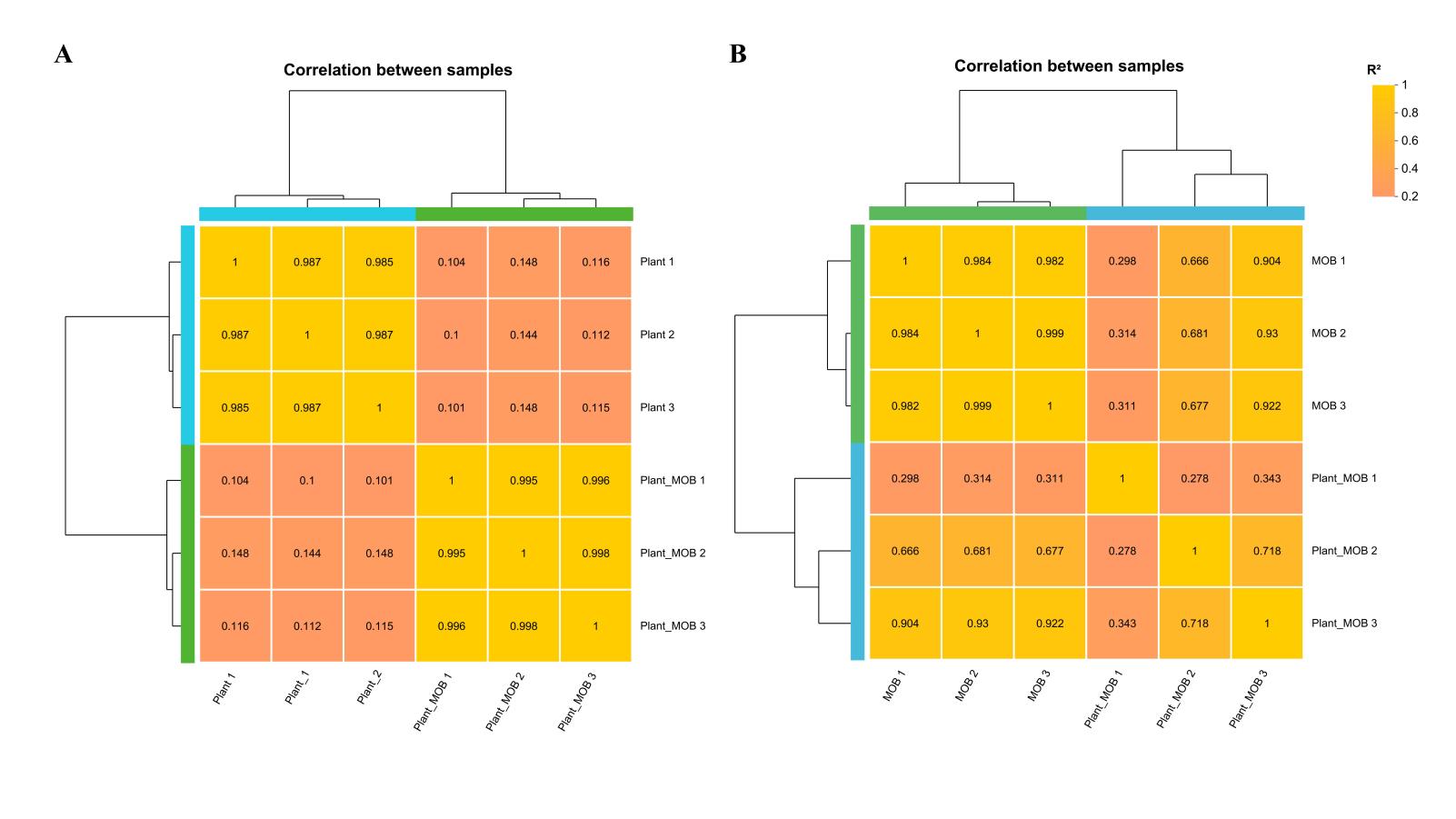


Fig. S2. Inter-sample correlation heatmap (n = 3)

(A) Correlation test between spinach samples; (B) Correlation test between *Exiguobacterium acetylicum* 4-3-1 samples.

The analyzed samples included *E. acetylicum* 4-3-1, control spinach, and spinach co-cultured with *E. acetylicum* 4-3-1 for a duration of 25 days cultured with 10.5 mg/kg of cadmium (Cd), followed by dual transcriptome sequencing (dual RNA-seq) analysis.


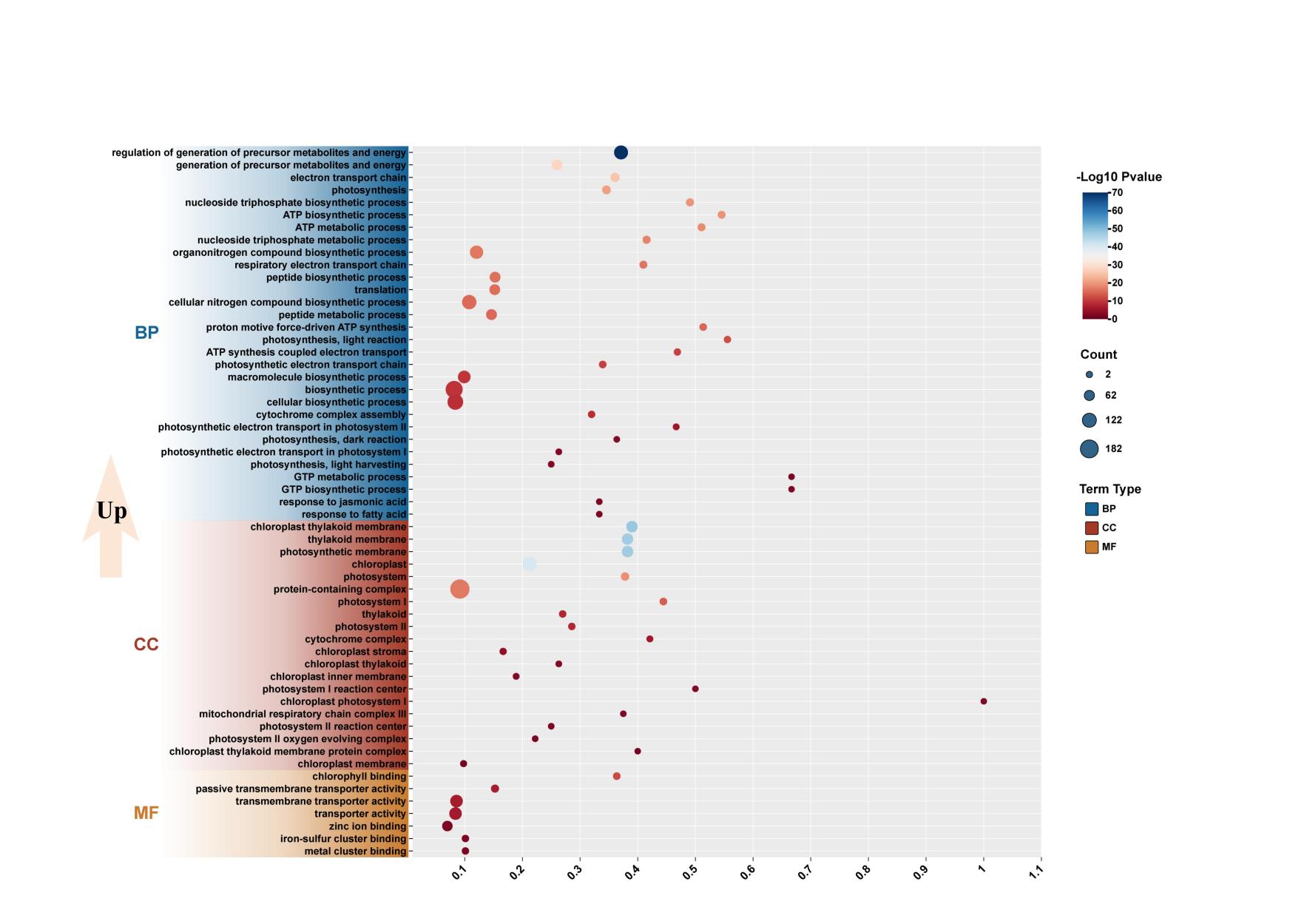


Fig. S3. Functional enrichment of upregulated genes related to cadmium stress based on GO database (n = 3).

The analyzed samples included control spinach and spinach co-cultured with *E. acetylicum* 4-3-1 for a duration of 25 days cultured with 10.5 mg/kg of cadmium (Cd), followed by dual transcriptome sequencing (dual RNA-seq) analysis.


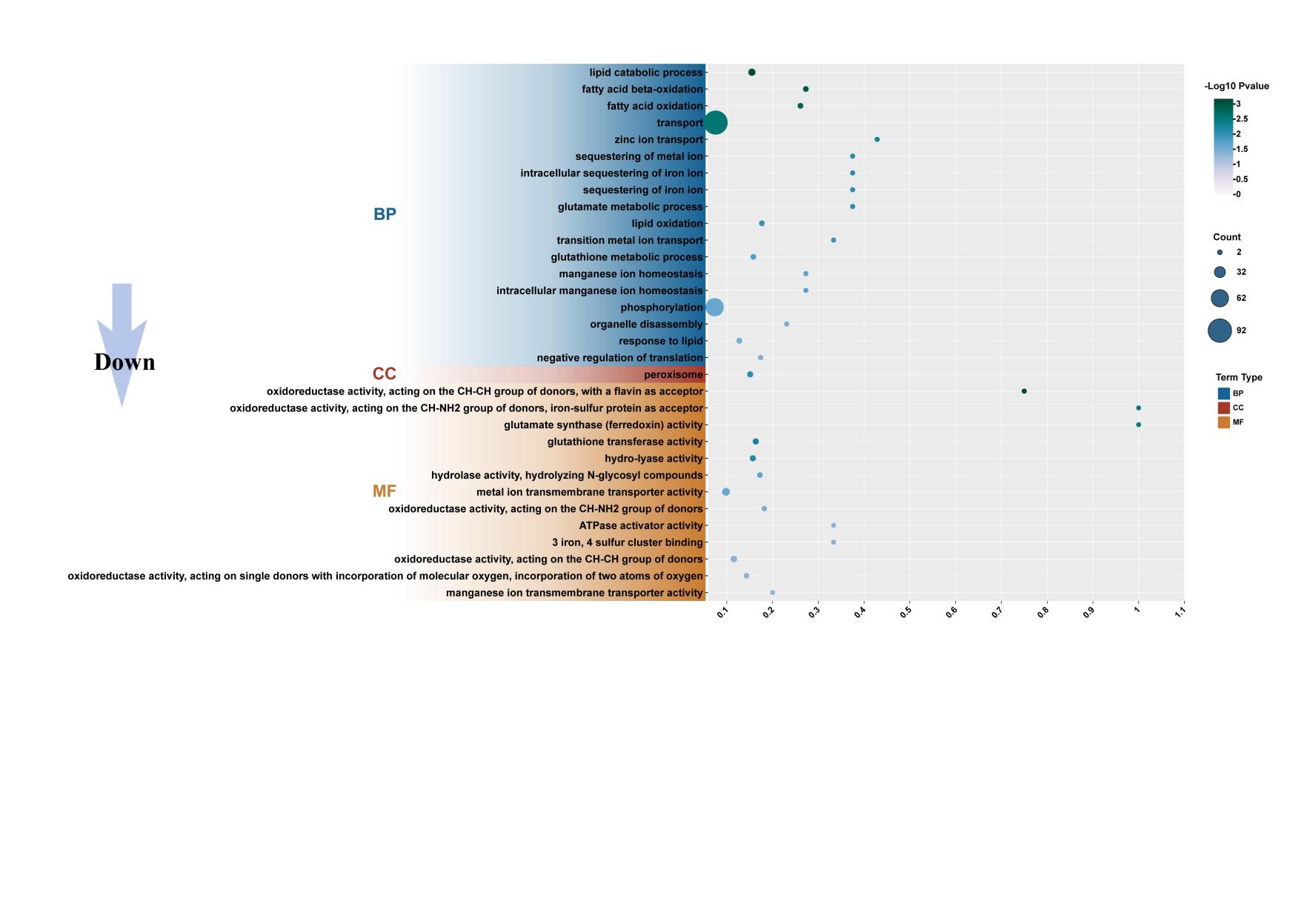


Fig. S4. Functional enrichment of down regulated genes related to cadmium stress based on GO database (n = 3).

The analyzed samples included control spinach and spinach co-cultured with *E. acetylicum* 4-3-1 for a duration of 25 days cultured with 10.5 mg/kg of cadmium (Cd), followed by dual transcriptome sequencing (dual RNA-seq) analysis.


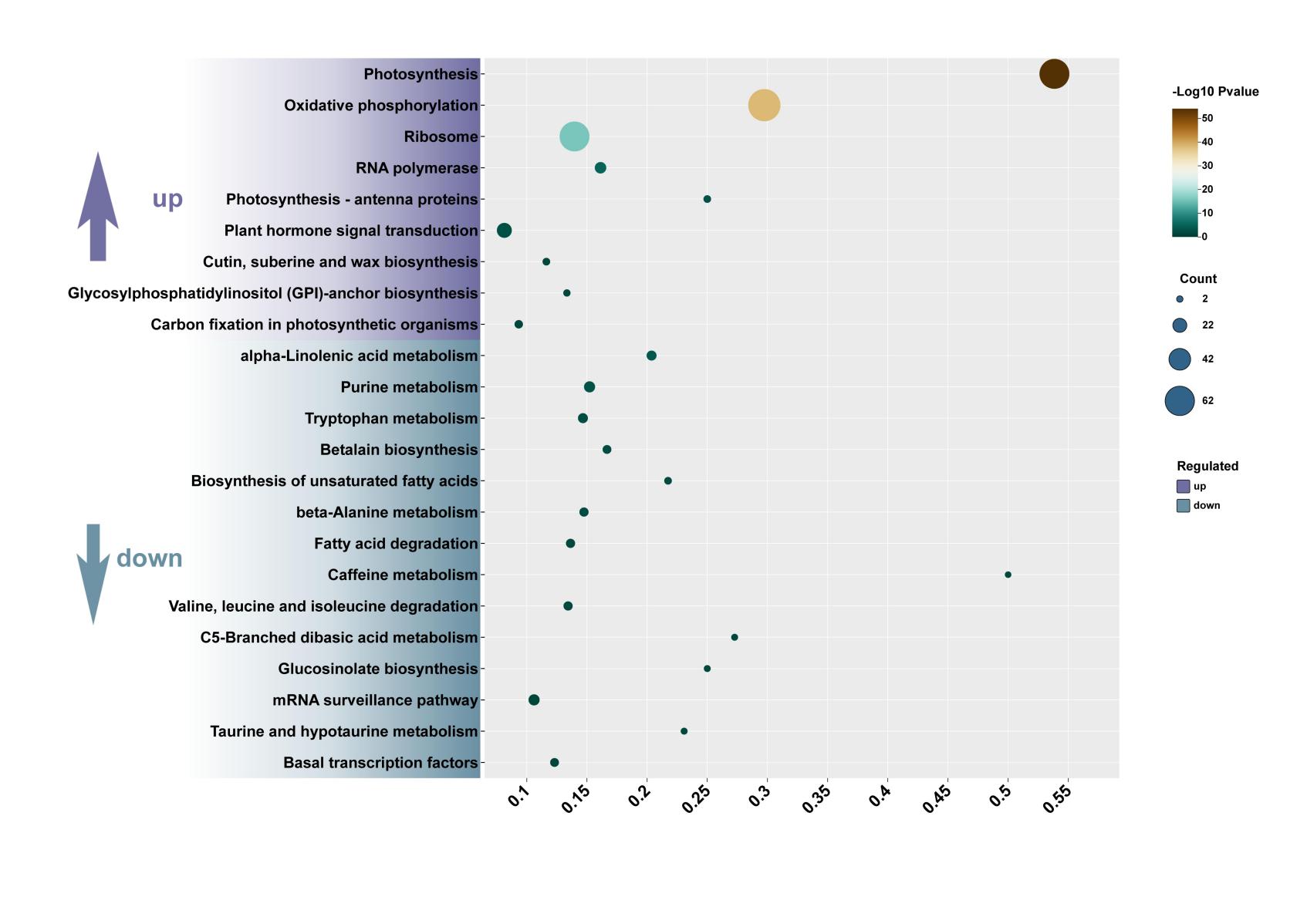


Fig. S5. Functional enrichment of differentially expressed genes related to cadmium stress based on KEGG database (n = 3).

The analyzed samples included control spinach and spinach co-cultured with *E. acetylicum* 4-3-1 for a duration of 25 days cultured with 10.5 mg/kg of cadmium (Cd), followed by dual transcriptome sequencing (dual RNA-seq) analysis.


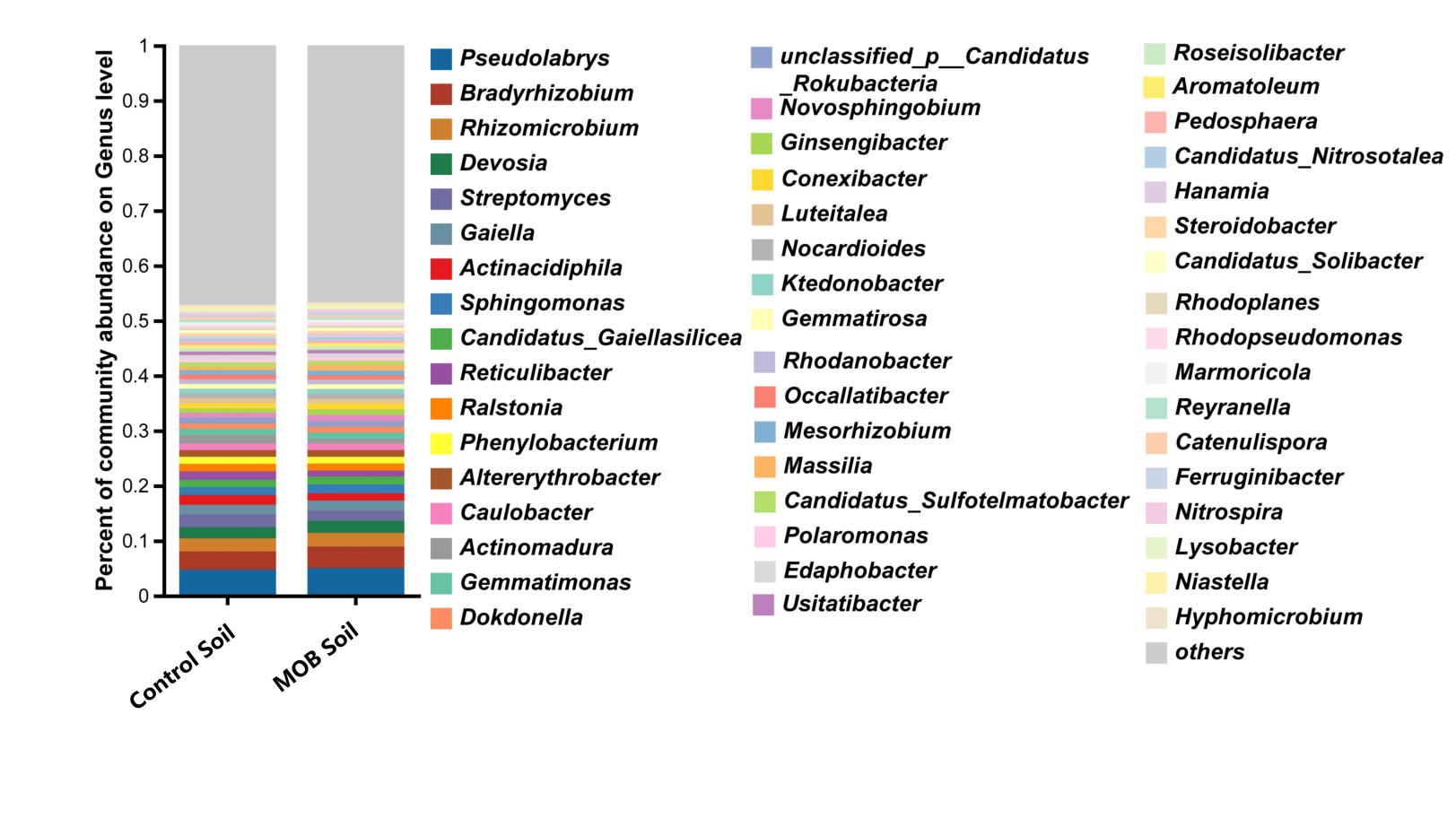


Fig. S6. Genus level composition of microbial communities in the rhizosphere soil under Cd stress (n = 3)

The experimental samples consisted of the rhizosphere soil of control group spinach and spinach co-cultivated with *E. acetylicum* 4-3-1 for 25 days cultivated with 10.5 mg/kg Cd, followed by metagenomic sequencing of the samples.


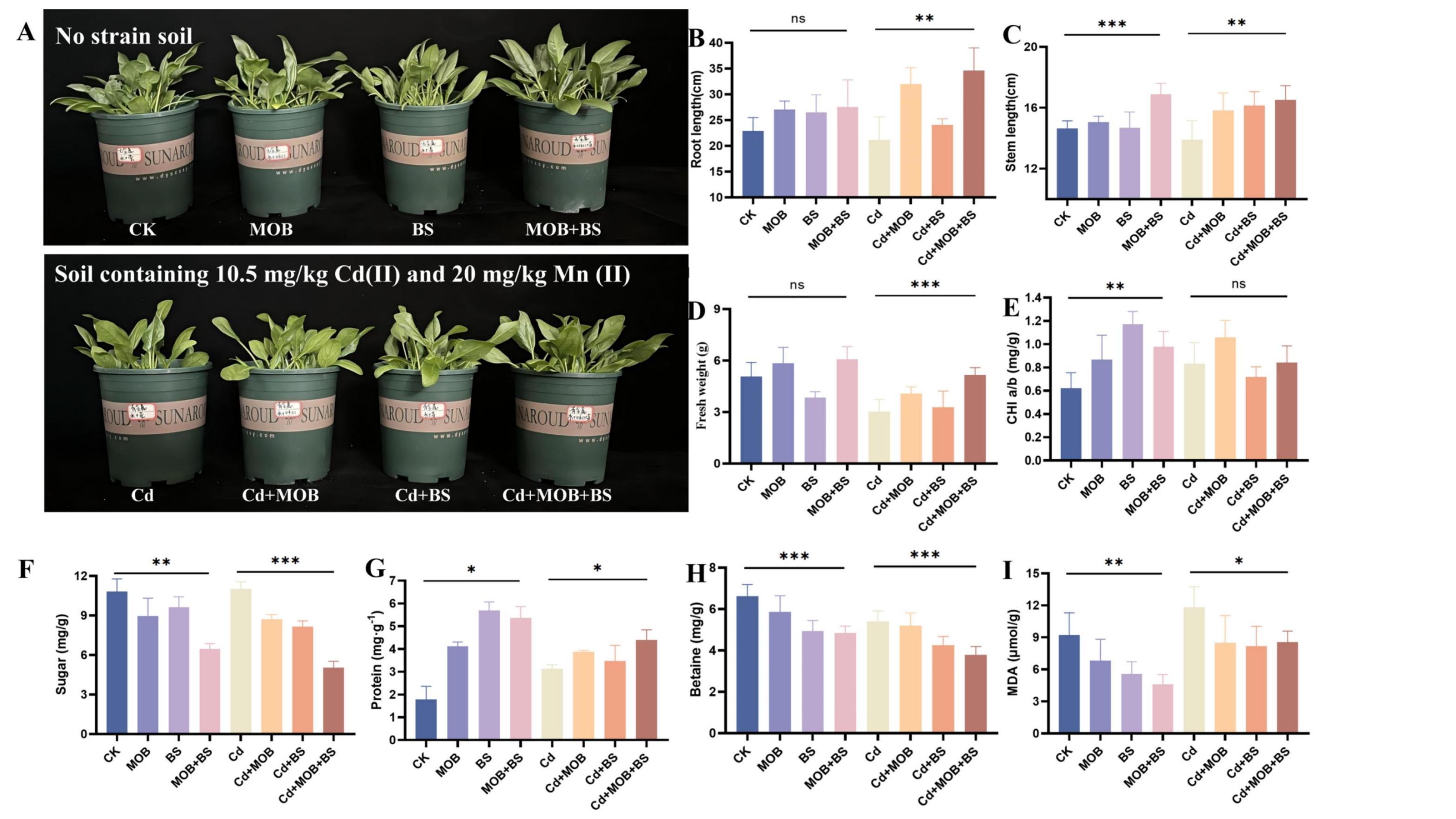


Fig. S7. Photos of spinach collection. CK, MOB, BS, and MOB+BS represent spinach in the control group, the group inoculated with *E. acetylicum* 4-3-1, the group inoculated with *Bacillus subtilis*, and the group with a synthetic community constructed with the two strains at a volume ratio of 1:1, respectively, set up in normal culture soil; Cd, Cd+MOB, Cd+BS, and Cd+MOB+BS represent spinach in the control group, the group inoculated with *E. acetylicum* 4-3-1, the group inoculated with *Bacillus subtilis*, and the group with a synthetic community constructed with the two strains at a volume ratio of 1:1, respectively, set up in cadmium-contaminated soil.


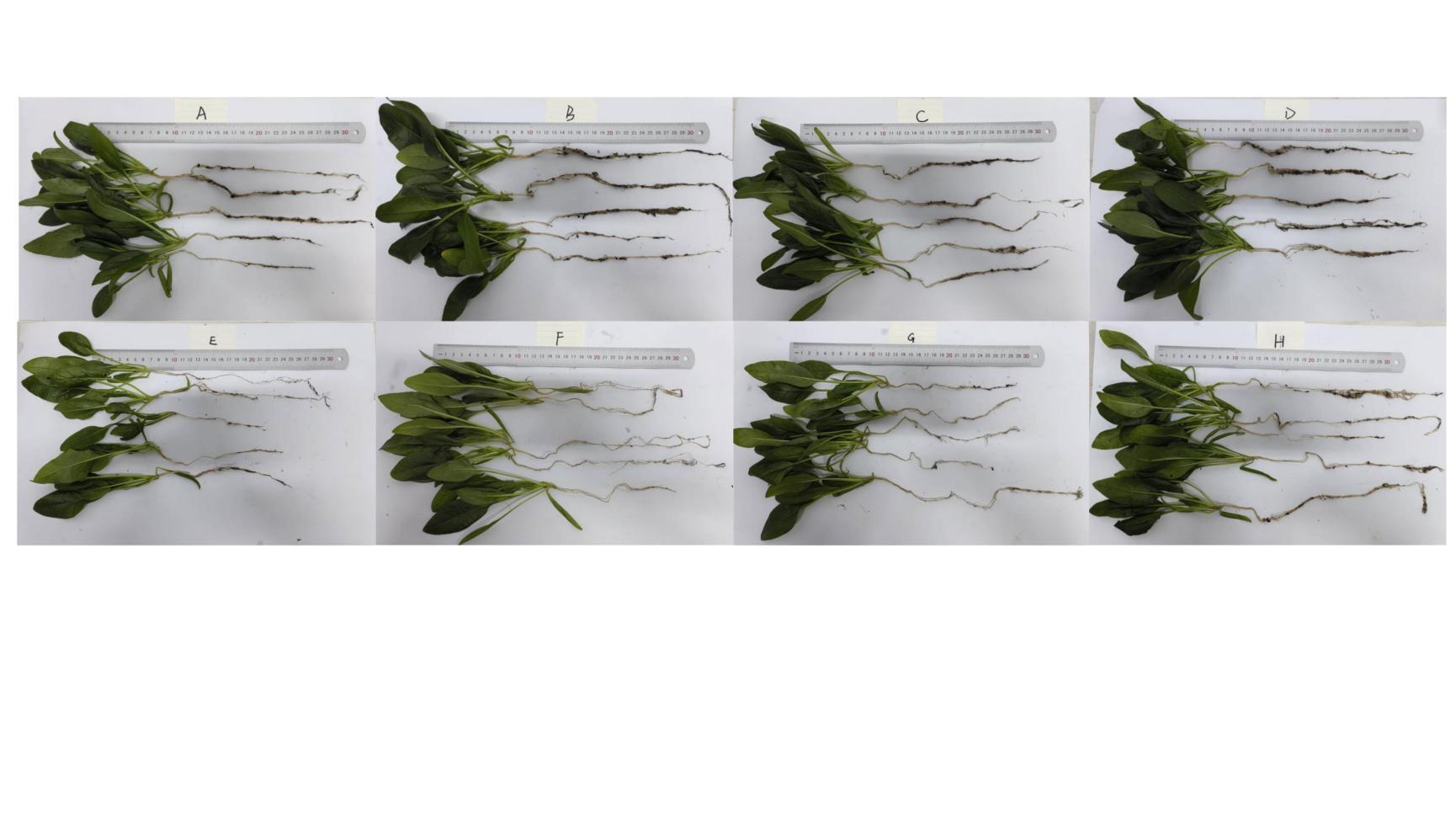


Fig. S8. Phenotypic observation of spinach. Groups A-D represent the control group, the group inoculated with *E. acetylicum* 4-3-1, the group inoculated with *Bacillus subtilis*, and the group with a synthetic community constructed with the two strains at a volume ratio of 1:1, respectively, under normal cultivation soil conditions. Groups E-H represent the control group, the group inoculated with *E. acetylicum* 4-3-1, the group inoculated with *Bacillus subtilis*, and the group with a synthetic community constructed with the two strains at a volume ratio of 1:1, respectively, under cadmium-contaminated soil conditions.
